# Development of a RAD-Seq Based DNA Polymorphism Identification Software, AgroMarker Finder, and Its Application in Rice Marker-Assisted Breeding

**DOI:** 10.1371/journal.pone.0147187

**Published:** 2016-01-22

**Authors:** Wei Fan, Jie Zong, Zhijing Luo, Mingjiao Chen, Xiangxiang Zhao, Dabing Zhang, Yiping Qi, Zheng Yuan

**Affiliations:** 1 State Key Laboratory of Hybrid Rice, Shanghai Jiao Tong University–University of Adelaide Joint Centre for Agriculture and Health, School of Life Sciences and Biotechnology, Shanghai Jiao Tong University, Shanghai, China; 2 Novel Bioinformatics Company, Shanghai, China; 3 Key Laboratory of Crop Marker-Assisted Breeding of Huaian Municipality, Jiangsu Collaborative Innovation Center of Regional Modern Agriculture and Environmental Protection, Huaiyin Normal University, Jiangsu, China; 4 Plant Genomics Center, School of Agriculture, Food and Wine, University of Adelaide, Waite Campus, Urrbrae, South Australia, Australia; 5 Department of Biology, East Carolina University, Greenville, North Carolina, United States of America; Louisiana State University Agricultural Center, UNITED STATES

## Abstract

Rapid and accurate genome-wide marker detection is essential to the marker-assisted breeding and functional genomics studies. In this work, we developed an integrated software, AgroMarker Finder (AMF: http://erp.novelbio.com/AMF), for providing graphical user interface (GUI) to facilitate the recently developed restriction-site associated DNA (RAD) sequencing data analysis in rice. By application of AMF, a total of 90,743 high-quality markers (82,878 SNPs and 7,865 InDels) were detected between rice varieties JP69 and Jiaoyuan5A. The density of the identified markers is 0.2 per Kb for SNP markers, and 0.02 per Kb for InDel markers. Sequencing validation revealed that the accuracy of genome-wide marker detection by AMF is 93%. In addition, a validated subset of 82 SNPs and 31 InDels were found to be closely linked to 117 important agronomic trait genes, providing a basis for subsequent marker-assisted selection (MAS) and variety identification. Furthermore, we selected 12 markers from 31 validated InDel markers to identify seed authenticity of variety Jiaoyuanyou69, and we also identified 10 markers closely linked to the fragrant gene *BADH2* to minimize linkage drag for Wuxiang075 (BADH2 donor)/Jiachang1 recombinants selection. Therefore, this software provides an efficient approach for marker identification from RAD-seq data, and it would be a valuable tool for plant MAS and variety protection.

## Introduction

Rice (*Oryza sativa* L.) is one of the most important crops, which feeds more than half of the world’s population [1, 2]. To meet the demand for feeding the growing population, rice production has to be greatly increased [2, 3]. Furthermore, due to better living standards, rice varieties with high eating and cooking quality are heavily emphasized during breeding selection processes, as they are key factors in attracting consumers and determining grain prices [4, 5]. In facing these challenges, new plant breeding technologies have to be developed [6, 7]. Conventional rice breeding methods mainly depend on phenotypic selection, a process strongly impacted by environmental factors, genotype factors, and interactions among them [1]. It might require 10–15 years to complete a variety breeding program from initiation to varietal release [1, 8], which is tedious and time consuming. Compared to conventional breeding, DNA polymorphism based marker-assisted selection (MAS) is more reliable and efficient, providing better selection strategies [9, 10] which can accurately select target genes in early generations and avoid the transfer of undesirable or deleterious genes. Recently, many rice varieties have been selected based on the MAS strategy [11–14], which opens up a new prospect for genetic improvement in rice.

Molecular marker-based technologies have been developed from hybridization-based, such as restriction fragment length polymorphism (RFLP) [15, 16], to PCR-based procedures, such as random amplified polymorphic DNA (RAPD) [17], simple sequence repeat (SSR) [18, 19] and amplified fragment length polymorphism (AFLP) [20, 21]. All of these markers have been widely used in species identification, phylogenetic analysis, genetic mapping, MAS, and so on [22]. But it takes a long time to determine the genetic or physical distance between markers and target loci before its application. Therefore, with the reduction of sequencing cost, more and more attention has been paid to the detection of genome-wide, high throughput sequencing-based molecular markers [23]. Several studies have reported the development of genome-wide markers in rice. For example, array-based resequencing technology has been used to discover genome-wide SNPs across a 100 Mb fraction of Nipponbare genome for 20 diverse varieties [24]. However, arrays are not able to detect unknown mutations or uncover structural DNA changes, such as translocations and inversions [25, 26]. Next-generation sequencing-based approaches can overcome these shortcomings. For example, through complete sequencing of 150 rice recombinant inbred lines (RILs), a total of 1,493,461 SNPs have been detected for recombination breakpoint determination and quantitative trait loci (QTL) analysis [27]. Later, based on whole genome resequencing technology, 2,819,086 DNA polymorphisms have been discovered between six elite *indica* rice inbreds and Nipponbare [28] and 1,154,063 DNA polymorphisms have been detected between a Korean rice accession and Nipponbare to estimate sequence diversity [29]. In addition, large amount of SNPs and InDels of three rice cultivars with contrasting drought and salinity stress responses have been identified to understand the genetic basis of phenotypic differences [30]. More recently, the 3,000 Rice Genomes Project provides new opportunities for rice research, which has completed sequencing of 3,000 rice genomes for revealing the genomic diversity across the world’s rice germplasm collections [31]. However, even though next-generation sequencing (NGS) platform can produce billions of DNA sequence data with greater resolution and accuracy [32], costs restrict its practice for some applications, especially in those wild germplasms and locally adapted varieties.

Recently, the restriction-site associated DNA (RAD) sequencing (RAD-seq) method was developed based on the NGS platform, further reducing the research costs. It only acquires the sequences adjacent to a set of particular restriction enzyme recognition sites so as to reduce the representation of a genome [33, 34]. Thus, compared to whole genome sequencing, the RAD-seq seems to be more flexible (with the choice of different restriction enzymes to control the marker density) and cost-effective. Besides, RAD-Seq technology can apply to species with no or limited genome sequence information [35]. The application of the RAD-seq technology facilitates the discovery of large volumes of polymorphism data across the genome, genetic mapping [34, 36, 37], genetic map construction [38–41], evolutionary studies [40, 42] and MAS [43]. However, drawbacks of the RAD-seq technology also exist. For example, the high probability of sequencing errors resulted from the NGS technology [44] and the presence of polymorphisms within the restriction site make it difficult to detect allelic polymorphisms [45]. In addition, sequencing data analysis is also a big challenge [46]. Although there are many softwares developed for marker identification [47], most of them are not easy to use because they require bioinformatics background. In addition, these softwares are often independent of each other so that compatibilities among them must to be considered to accomplish the whole analysis.

To simplify the RAD-seq data analysis, we developed an integrated software named AgroMarker Finder (AMF), which combines external tools with self-developed programs and provides a user-friendly graphical interface, making it more accessible to users. To demonstrate the application of AMF in rice breeding and functional studies, a set of 90,743 genome-wide markers had been detected between rice *Indica* variety JP69 and *japonica* variety Jiaoyuan5A using this software. Both rice varieties are parents for a new authorized hybrid rice variety, Jiaoyuanyou69, which shows heterosis. In addition to analyzing the distribution and functional relevance of these markers, a subset of 82 SNPs and 31 InDels were validated for their close linkages to 117 important agronomic trait genes, and 10 of the 31 InDels have been used to identify seed authenticity of the Jiaoyuanyou69 variety. Finally, by running AMF, 10 markers closely linked to the fragrance gene *BADH2* were selected to minimize linkage drag for MAS. In the F7 generations of Wuxiang075 (donor)/Jiachang1 cross, both linkage analysis and phenotypic analysis on fragrance confirmed that we obtained 8 lines in Jiachang1 background carrying *BADH2* chromosomal segment introgressed from Wuxiang075. Refinement of the intervals carrying the *BADH2* gene in 8 lines revealed that the shortest donor segment for the introgression of *BADH2* was approximately 20.75 cM. Therefore, this study developed and validated a powerful software, AMF, for RAD-seq based genome-wide marker identification. Its user-friendly interface has been demonstrated in rice. It could be broadly used for MAS in other plants as well.

## Materials and Methods

### Plant material

All plant materials including JP69, Jiaoyuan5A, Jiaoyuanyou69, Wuyungeng7, Jiachang1, Wuxiang075, and the F7 population from a cross between Jiachang1 and Wuxiang075 were grown in the paddy field or greenhouse of Shanghai Jiao Tong University at Shanghai in China.

### DNA extraction, library preparation and sequencing

Genomic DNA was extracted from 100 mg fresh young leaves (from pooled samples for each variety) using the CTAB method [48]. One microgram DNA was digested for 10 min at 65°C in a 30 μl reaction with 20 units (U) of TaqαI (New England Biolabs). T4 ligase was used to ligate the adapters to DNA fragments at 22°C for 60 min, then the enzyme was heat-inactivated at 65°C for 30 min. TaqαI was inactivated by adding trichloromethane into the reaction system before selection of DNA fragments. Paired-end sequencing was performed on an Illumina HiSeq2000 after the selection of 400–600 bps adaptors-ligated and PCR-amplified DNA fragments on gel. All these experiments were performed by the Beijing Genomics Institute (BGI). Sequencing data have been deposited at the Sequence Read Archive (SRA) under the accession number SRP052892.

### AgroMarker Finder analysis pipeline

**Fig 1:**
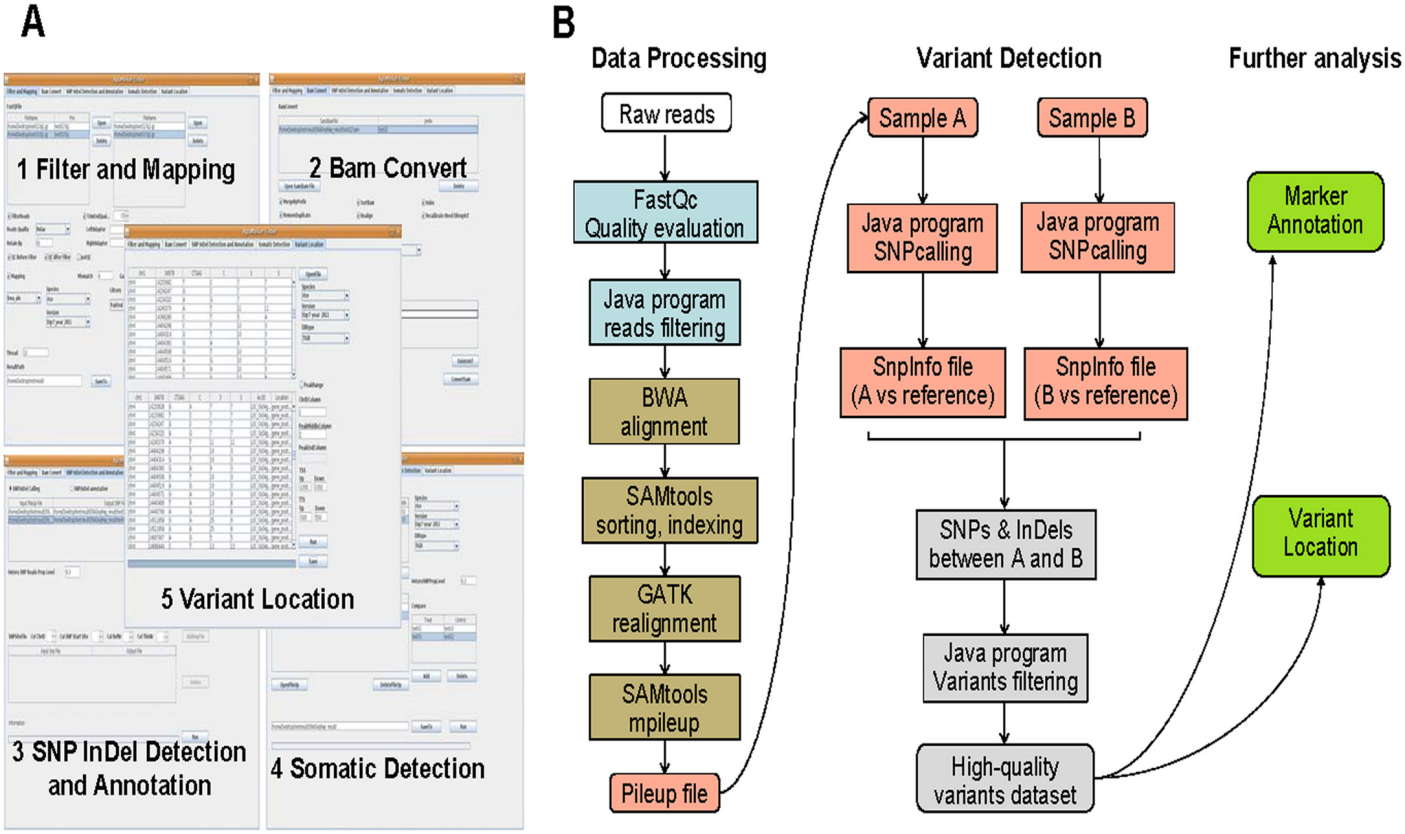
Development of AgroMarker Finder (AMF). (A) Graphical User Interface of AgroMarker Finder. (1) Filter and Mapping module for quality assessment, filtering and mapping of data. (2) Bam Convert module for manipulating alignments in BAM or SAM format, including merging, filtering multiple mapped reads, sorting, indexing, removing duplicates, realigning and recalibrating. (3) SNP InDel Detection and Annotation module for SNP/InDel calling and annotation. (4) Somatic Detection module for variant discovery between two samples. (5) Variant Location module for locating the region of mutation. (B) Analysis pipeline showing procedures for RAD-seq data processing based on AMF. Data processing (shown in dark grey) trims and filters raw reads to remove low-quality bases and reads. Quality of raw data and filtered data are evaluated by FastQC. Filtered data are aligned against a reference genome using BWA. Uniquely mapped reads are sorted and indexed by SAMtools. Afterwards data are realigned by GATK. Then, SAMtools is used to generate pileup format files for each sample (in yellow). These pileup files are input for variant detection and filtering (in orange and grey) using the Java program with optional parameters. High-quality markers are annotated and located to linked genes (in blue) based on the Java program.

The procedure of the analysis is presented in Fig 1B. Filtering of Illumina raw sequencing reads was preformed based on a Java program. Firstly, 84 bp raw sequencing reads were trimmed from both 3’ and 5’ ends (bases with Q<20 [36, 38, 49]). Only trimmed reads up to 50 bp were retained. Then, low-quality reads were discarded with the relax standard. The retained reads were aligned to the reference genome (Nipponbare, Rice Genome Annotation Project) using Burrows-Wheeler Aligner (BWA) with parameters mismatch = 4, gap length = 20 [50]. To avoid the influence of false mapped sequences on the accuracy of the final results, only those mapping to a unique position in the genome were reserved from the BWA results. After sorting and indexing based on SAMtools [51], the realignment analysis was performed based on GATK [52]. Then, SAMtools was used to summarize the base calls of aligned reads to the reference genome. The output files can be directly used to detect variants based on our self-developed SNP InDel Detection and Annotation module and Somatic Detection module (see AgroMarker Finder Manual). In this study, HeteroSNPPropLevel was set to 0.3 to capture more candidate sites. Finally, in order to improve the validity of variants, we filtered variant data generated by Somatic Detection application in accordance with the following conditions: (1) Only positions covered by at least eight reads in one sample and none in the other were reserved (Figure A in S1 File); (2) All variants with intervals less than 10 bp were eliminated.

Gene annotation of variants was also accomplished by AMF. Rice genome annotation file in GFF3 format was retrieved from Rice Genome Annotation Project. All 90,743 polymorphic sites were assigned to specific chromosome regions surrounding these sites for predicting their structures and functions. The output file described the SNP location (exons, introns, intergenic regions, 5’UTRs, 3’UTRs or intergenic regions) and gene functional relevance (synonymous or nonsynonymous).

### Sequencing validation of SNPs and InDels

By application of AMF analysis, a subset of 121 markers (Table A in S1 File), which are tightly linked to 117 important agronomic trait genes with genetic distance less than 5 cM, was identified by Sanger sequencing to evaluate the accuracy. Flanking primers were designed by Primer 3.0 software ([53]). Each PCR reaction had 1 × PCR buffer, 0.2 mM dNTP, 0.4 μM each of forward and reverse primers (Table B and Table C in S1 File), 20 ng of each genomic DNA, and 1.25 unit of Taq DNA polymerase, in a final volume of 25μl. The cycling conditions were as follows: denaturing of DNA at 94°C for 5 min, 35 cycles of 30 s at 94°C,annealing at 54°C for 30 s, 40s at 72°C, and a final extension at 72°C for 5 min. The PCR products were resolved by electrophoresis with 2% agarose gels in 0.5 × TBE and stained by GelRed. Then, verified fragments were analyzed using the Sanger sequencing method (Beijing Genomics Institute).

### Rice variety identification

The validated 121 markers (Table A in S1 File) were used for variety identification. Twelve markers were selected and 9 samples were collected for rice variety identification, including JP69, Jiaoyuan5A, Jiaoyuanyou69, Wuyungeng7 and 5 blind samples. After simultaneous amplification of these 9 samples, PCR products were resolved using the MultiNA microchip electrophoresis system (Shimadzu Corporation, Japan). Similarity was confirmed by comparing the polymorphism of amplified bands from 5 blind samples to that of Jiaoyuanyou69.

### MAS on the *BADH2* gene

For performing MAS on the *BADH2* gene, genomic DNA of the donor variety Wuxiang075 and the recipient variety Jiachang1 were extracted for RAD sequencing as described above. In the F1 generation of Wuxiang075/Jiachang1 cross, plants with fragrance were retained. By application of AMF, 10 markers that are adjacent to *BADH2* were selected from 7922 markers in the library to minimize linkage drag (Table C in S1 File). The selected 5 lines containing the desired *BADH2* allele were self-crossed and further tested in the next generations to confirm the recombination.

## Results

### Development of AMF

Currently, many tools are available for NGS data analysis [47]. However, most of them only possess individual functions and provide command line interfaces, which require bioinformatics expertise. Moreover, concerted interoperation of different softwares for a complete analysis is arduous. For rapid and accurate marker identification based on RAD-seq data, we developed a software package, AMF (http://erp.novelbio.com/AMF), which has a graphical user interface (GUI) that visualizes the data analysis process and makes it accessible to general users (Fig 1A). It provides a versatile computational pipeline for quality assessment, filtering, mapping, variant detection, annotation and variant location through five modules: Filter and Mapping, Bam Convert, SNP InDel Detection and Annotation, Somatic Detection and Variant Location (Fig 1).

In Filter and Mapping, FastQ formatted RAD data or whole genome sequencing data are allowed to import for filtering to remove low quality reads or trim adaptor sequences of low quality bases. It provides four different filtering criteria for sequencing reads: (I) Strict standard. Reads showing 7 percent bases with Q<10 or 7 percent with Q<13 or 15 percent with Q<20 are discarded. (II) Moderate standard. Low-quality reads showing 10 percent bases with Q<10 or 14 percent with Q<13 or 20 percent with Q<20 are removed. (III) Relax standard. Low-quality reads showing 15 percent bases with Q<10 or 20 percent bases with Q<13 are discarded. (IV) NotFilter. All reads will be kept without filtering. These filtering criteria can be freely combined with TrimEndQuality (Q score) to gain a broad range of filtering criteria. For quality checking, FastQC (http://www.bioinformatics.babraham.ac.uk/projects/fastqc/) was employed before and after filtering. Then, filtered reads are mapped to the reference genome based on Burrows-Wheeler Alignment (BWA), which is a fast and accurate short read alignment tool [50]. Besides, Bowtie 2, a memory-efficient short read aligner, is also integrated in the AMF application package to meet different needs [54].

In Bam Convert, both Sequence Alignment/Map files and Binary Alignment/Map (BAM) files can be used. Files with the same prefix will be merged firstly by MergeByPrefix and SAM formatted files will be converted to BAM files directly. Then, uniquely mapped reads can be reserved by FilterMultipleMappedReads. Next, BAM files are sorted and indexed based on Sequence Alignment/Map tools (SAMtools) [51]. After that, ‘samtools rmdup’ command line can be used to remove potential PCR duplicates, and The Genome Analysis Toolkit (GATK) is employed to perform realignment analysis and recalibration analysis, which could reduce mapping errors and eliminate some false positive SNPs [52]. We then use SAMtools to summarize the base calls of aligned reads to the reference genome.

The generated Pileup files can be directly used to detect SNPs/InDels in SNP InDel Detection and Annotation module. The strategy is as follow: mutant site (different from the reference genome) should be covered by at least 3 reads containing more than 2 mutant reads, and mutant ratio should exceed 20%. Based on different requests, mutant ratio can be defined autonomously to determine a mutant site. The output SnpInfo file records the coordinate and coverage information in a straightforward TXT format. The detected polymorphisms in SnpInfo files can then be located to the reference genome for investigating their distribution and functional relevance based on GTF (Gene Transfer Format) or GFF (General Feature Format) files by SNP/InDel annotation function.

In Somatic Detection, users should simultaneously input SnpInfo files and Pileup files to detect polymorphisms between two samples. One sample is regarded as Control group and the other is regarded as Treat group. For control group, site covered by at least 10 reads including less than 2 mutant reads (different from the reference genome) and containing less than 4% mutant ratio will be kept for further comparison. For Treat group, the filtering strategy for mutant sites is the same as in SNP InDel Detection and Annotation module. Then, the software will record polymorphisms by comparing mutant sites kept in Treat group to Control group. To obtain complete SNPs and InDels information between two samples, users should exchange the positions of two samples for comparison.

In Variant Location, based on GFF or GTF files, the region of mutations can be accurately located according to the chromosome and coordinate information of SNPs and InDels, and the output file provides users with detailed position information of polymorphisms. The packages and manual are publicly available at http://erp.novelbio.com/AMF/ and https://github.com/NovelBio-Bioinformatics-Company/NBCsoftware.

### Application of AMF in SNP/InDel detection

**Fig 2:**
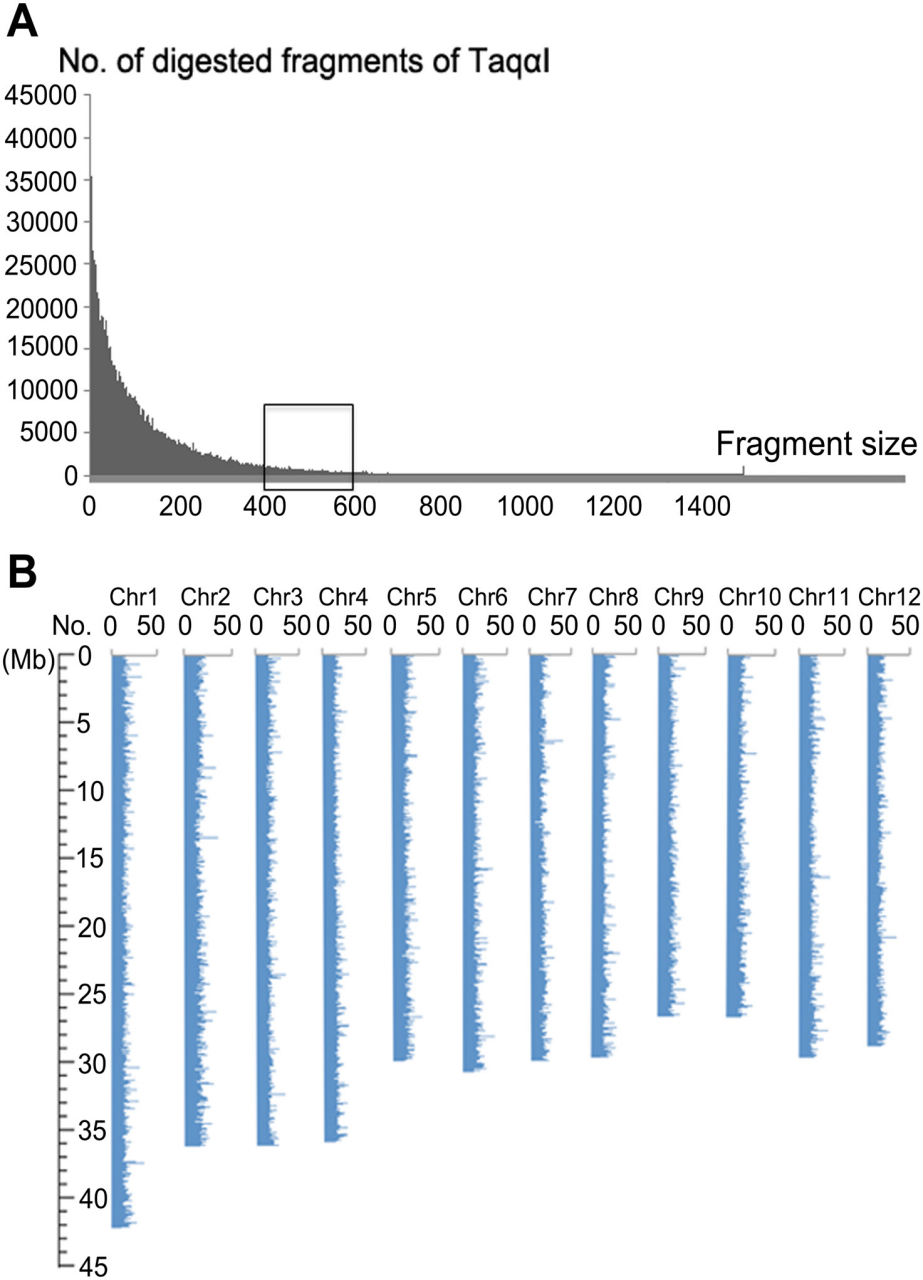
In-silico prediction of digested fragments of the rice genome. (A) In-silico digestion with TaqαI of rice genome, showing 130,940 digested fragments between 400–600 bp in size. The X axis indicates the length of digested fragments; The Y axis indicates the number of digested fragments. (B) Density distribution of TaqαI recognition sites in the rice genome. The X axis indicates twelve chromosomes; The Y axis indicates the number of restriction enzyme sites. The result showed the restriction digestion sites for TaqαI were evenly distributed in the rice genome.

Jiaoyuanyou69 is a newly authorized superior hybrid rice in Shanghai, which is the F1 generation from a cross of *indica* variety JP69 and *japonica* variety Jiaoyuan5A. Jiaoyuanyou69 shows high heterosis and its grain yield reaches 843.16kg/667m^2^. For the subsequent construction of near isogenic lines (NILs), functional genomic research and MAS, JP69 and Jiaoyuan5A were used to mine genome-wide polymorphic information by the RAD-seq technology. The RAD tags were generated on an Illumina HiSeq2000 sequencing machine. In order to ensure that sufficiently digested fragments were within the desired size range (400–600 bp), we performed in-silico prediction of restriction enzymes on Nipponbare genome (TIGR 7, http://rice.plantbiology.msu.edu/). The distribution of restriction enzyme sites and the length of digested fragments were calculated using a Java program (see AgroMarker Finder Manual). Among all the restriction enzymes, the digestion sites of TaqαI (T/CGA) are evenly distributed in rice genome, producing about 130,940 digested fragments between 400–600 bp in size (Fig 2), which met the required number of RAD tags for subsequent genome sequencing analysis (100,000–150,000) [38].

After sequencing, a total of 40,521,314 paired-end raw reads were collected, with 16,970,386 reads for JP69 and 23,550,928 reads for Jiaoyuan5A. To avoid sequencing errors, reads were then trimmed with Q<20 and filtered with the relax standard option. These retained reads were mapped to Nipponbare genome for screening uniquely mapped reads (Fig 1B). Finally, 22,517,142 high-quality reads were retained from the BWA results and used for further analysis. Among them, 8,893,480 reads were from JP69, and 13,623,662 reads were from Jiaoyuan5A (Table D in S1 File). After filtering (for detailed method, please see Experimental procedures), a total of 90,743 high-quality makers (82,878 SNPs and 7,865 InDels) were obtained between JP69 and Jiaoyuan5A. The SNP frequency detected by AFM is 0.2 per kb, and the InDel frequency is 0.02 per kb (Table 1).

**Table 1:** SNP/InDel frequency detected by AMF between JP69 and Jiaoyuan5A.

Chromosome	1	2	3	4	5	6	7	8	9	10	11	12	Total/Average
Number of InDels	1106	923	875	618	599	430	669	589	456	617	642	341	7865
Number of SNPs	9966	9414	8177	7420	6212	3786	6842	6941	5359	6910	7872	3979	82878
Density of InDel (per kb)	0.026	0.026	0.024	0.017	0.02	0.014	0.023	0.021	0.02	0.027	0.023	0.012	0.021
Density of SNP (per kb)	0.23	0.26	0.22	0.21	0.21	0.12	0.23	0.24	0.23	0.3	0.27	0.14	0.22

**Fig 3:**
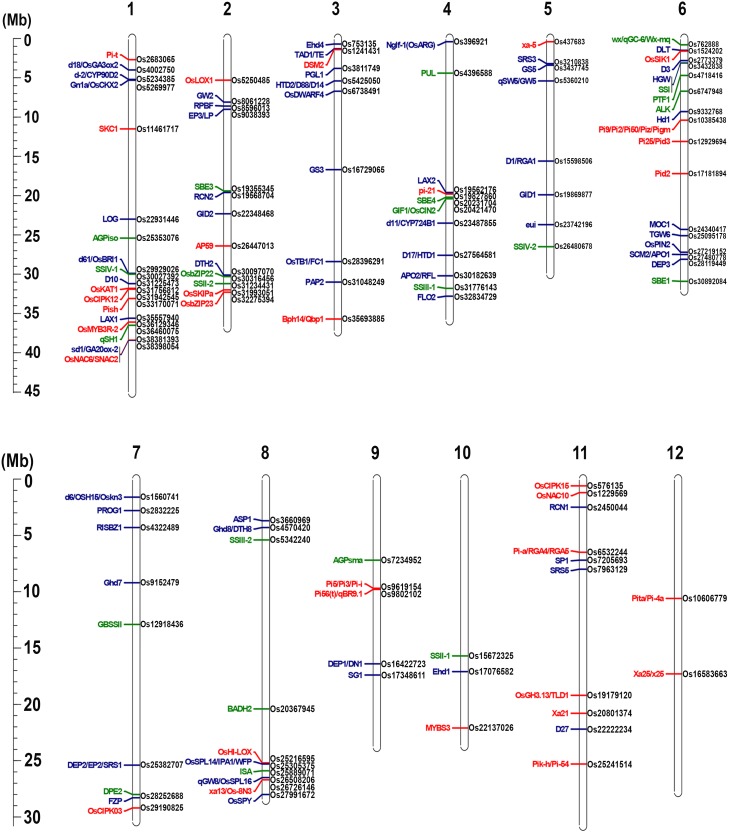
Identification of markers associated with rice agronomic genes between JP69 and Jiaoyuan5A. Distribution of rice agronomic genes and linked markers. Red represents disease resistance related genes; blue represents yield related genes; green represents quality related genes. Genes are labeled on the left of chromosomes; linked markers of each gene are labeled on the right. Those incorrect markers have been replaced, and all listed markers have been validated.

To verify the accuracy of these markers identified by AMF, a subset of 121 markers, which was closely linked to 117 important agronomic trait genes with genetic distance less than 5 cM, was verified by Sanger sequencing (Fig 3; Table A in S1 File) [47, 55]. The heterozygous and false calling sites were all included for error rate calculation. Finally, 113 markers consisting of 82 SNPs and 31 InDels were validated, suggesting a high accuracy of 93% (Figure B and Table B in S1 File).

### Characterization and annotation of SNPs and InDels

**Fig 4:**
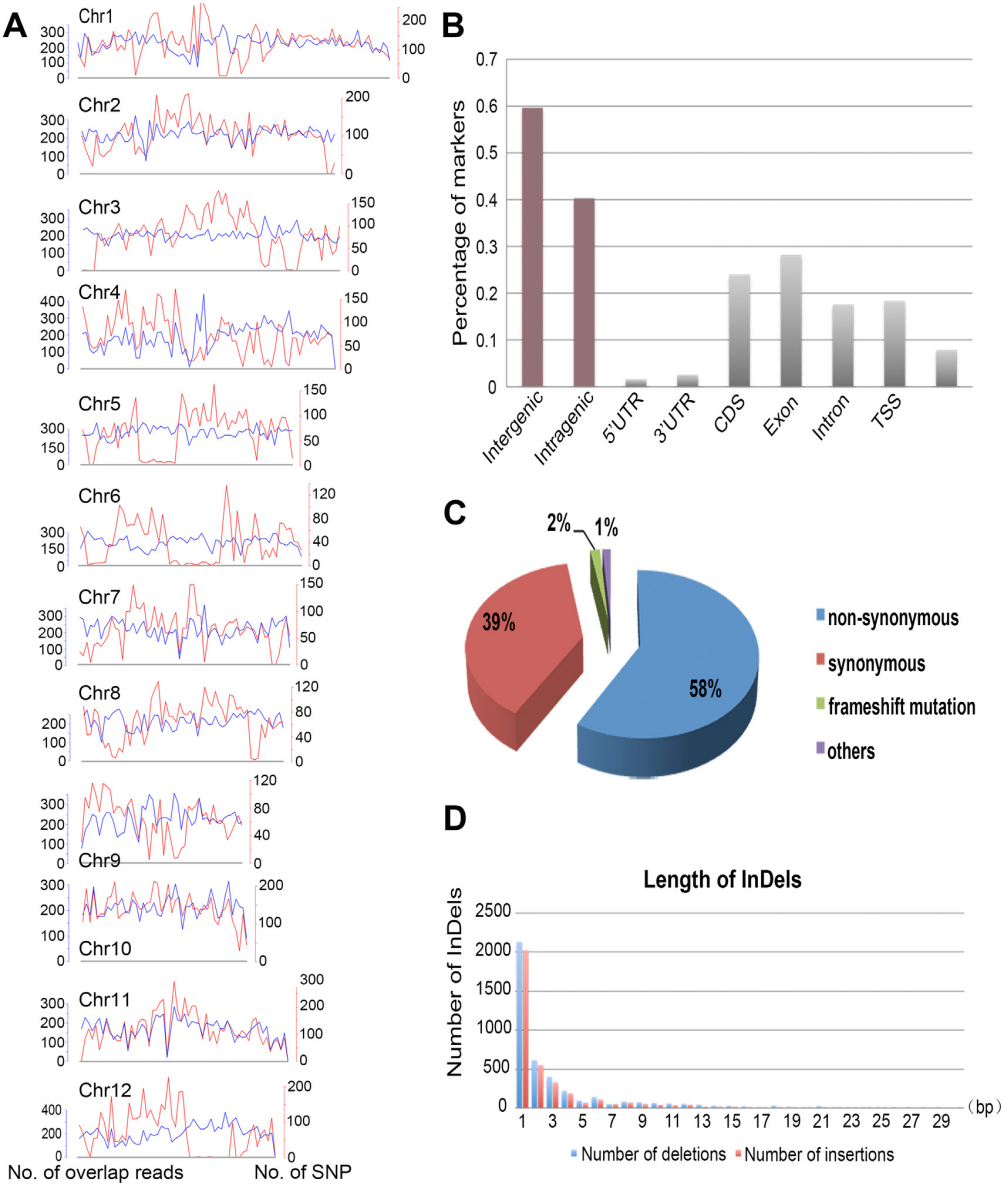
Marker distribution and annotation. (A) Distribution of the number of overlapping reads and SNPs between Jiaoyuan5A and JP69 along each chromosome in 500 kb windows. (B) Distribution of 90,743 markers in different genomic regions. The X axis indicates different genic regions; The Y axis indicates the number of markers. (C) Functional relevance of polymorphisms located in the coding regions. (D) Length of insertions and deletions. The X axis shows the length of insertions (shown in red) and deletions (shown in blue). The Y axis shows the number of insertions and deletions at each length.

The 90,743 high-quality markers were then located to the Nipponbare genome for investigation of their distribution and functional relevance. It was not surprising that all polymorphic sites were unevenly distributed on each chromosome (Fig 4A). Polymorphism-poor regions (<98 per Mb) were found in all chromosomes except chromosome 10, while polymorphism-rich regions (>491 per Mb) were observed in chromosomes 2, 3, 7, and 11. Those regions with low polymorphic density might be conserved that share a common ancestral origin between JP69 and Jiaoyuan5A in Chromsome 5, 6, and 12 (Fig 4A) [56]. Further analysis of the relevance between density of polymorphisms and chromosomal regions revealed that polymorphism-rich region on chromosome 7 and 11 contained both putative intergenic and transcribed regions, whereas those detected in chromosomes 2, 3, and 11 were in centromeric regions. This uneven distribution pattern of polymorphism sets has been reported in many rice cultivars [28, 57, 58], and there seems to be a selective mechanism in the process of domestication [58, 59]. We defined that transcription start site (TSS) regions and transcription terminate site (TTS) regions are located at position -1000 to +1000 from TSS and -500 to +500 from TTS, respectively. We found that more polymorphisms were present in intergenic regions (59.7%), which was similar in other rice varieties and plants [30, 60, 61]. The remaining polymorphisms (40.3%) were detected in genic region, of which 61.7% were observed in exonic regions and 52.4% in coding regions (CDS) (Fig 4B).

Further analysis of polymorphisms located in the coding regions revealed that 58% of these polymorphisms would introduce non-synonymous mutations. Among them, 3.9% polymorphisms would result in the introduction of a stop codon, producing premature proteins. And 0.07% of the polymorphisms would change the stop codon into non-stop codon, which causes the abnormal peptide synthesis (Fig 4C). Previous studies have demonstrated the importance of these genetic polymorphisms in regulation of gene expression and phenotypic traits of plant [62].

Then, we analyzed the length distribution of InDels between JP69 and Jiaoyuan5A. Among 7865 InDels, 4202 deletions were detected, the length of which ranged from 1 to 29 bp,and the number of insertions was 3663, the length of which was up to 30 bp (Fig 4D). It showed that most of the InDels (52.8%) were mononucleotide and 31.2% of which were 2 to 5 bp InDels. Only a few InDels (0.27%) were longer than 22 bp (6 insertion and 15 deletions), two thirds of which were located in centromere-specific retrotransposons and genes involved in processes such as sterol transport, flowering, polygalacturonase inhibition. Further studies on these large InDels would provide valuable insights into the genetic basis of phenotypic differences between these two rice varieties.

### Application of markers in rice variety identification

To identify seed authenticity of Jiaoyuanyou69, we selected 12 InDel markers (Table C in S1 File) from 31 validated InDels described above. Among 5 blind rice samples with similar morphological characteristics that cannot be distinguished by straightforward phenotypic examination, blind sample 1 always showed similar amplified bands with Jiaoyuanyou69 positive control (Figure C in S1 File), thus confirming the similarity between them. This result demonstrates that the markers identified by AMF could also be applied in variety registration and protection.

### Application of AMF in targeted breeding of the *BADH2* gene

**Fig 5:**
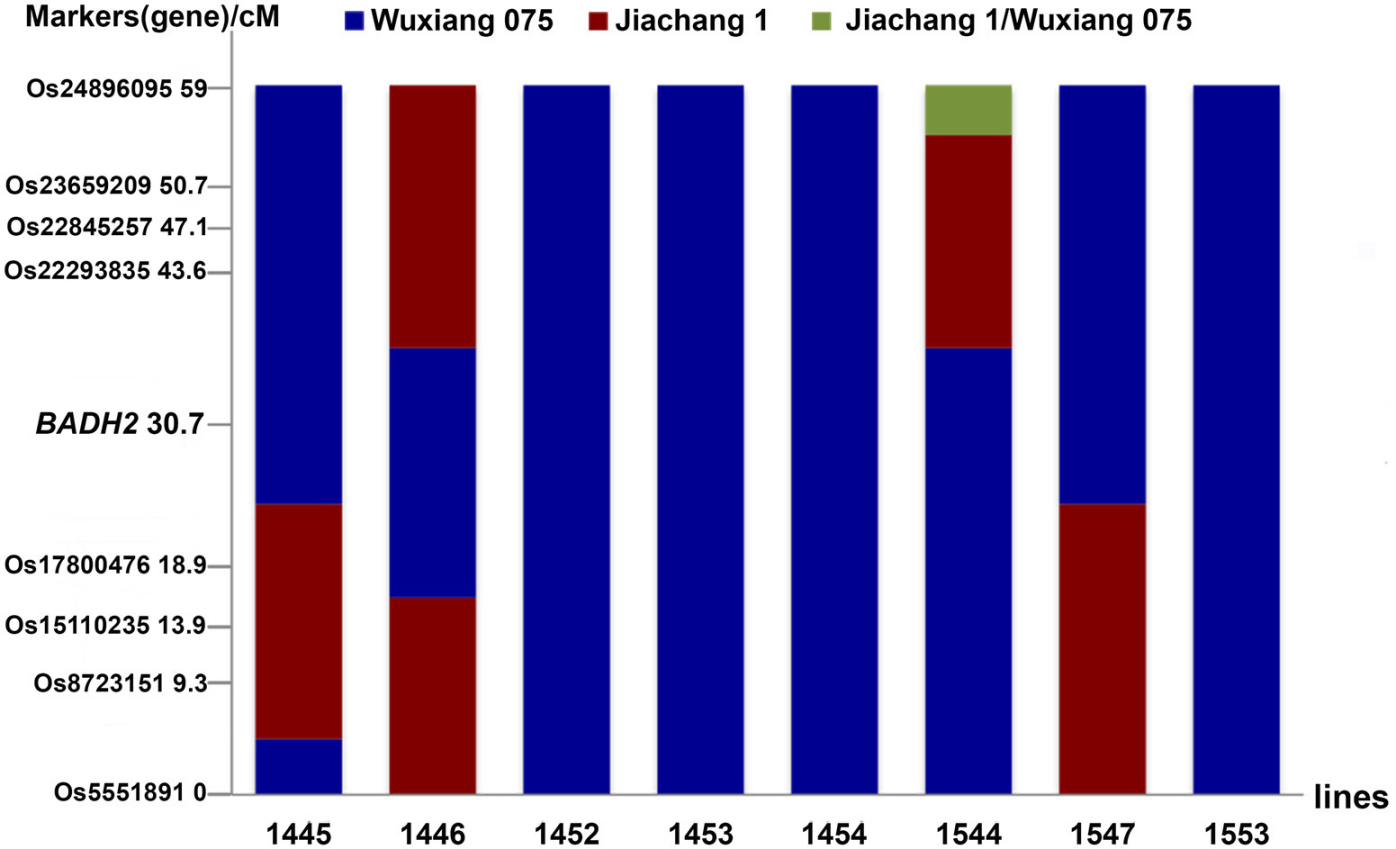
Analysis of genome introgression containing *BADH2* fragment in the F7 generations of Wuxiang075/Jiachang1 cross. The X axis shows the 8 lines selected based on two markers Os20105487 and Os20557750; The Y axis indicates the distance distribution of 8 markers for further analysis.

Fragrant rice is gaining popularity among consumers worldwide. Studies have indicated that 2-acetyl-1-pyrroline (2AP) is a potential flavor component giving rice distinctive fragrance, which is controlled by BADH2, a betaine aldehyde dehydrogenase that is responsible for aroma metabolism in fragrant rice varieties [63]. The donor parent Wuxiang075 produces rice with tempting fragrance while Jiachang1 has high yield. To perform MAS on the *BADH2* gene in the progeny of Wuxiang075/Jiachang1 backcross, RAD-seq data of Wuxiang075 and Jiachang1 were analyzed by AMF. In total, 7922 markers were obtained, and two markers, (Os20105487 and Os20557750) adjacent to *BADH2* (less than 1.5 cM) were selected to perform target locus selection (Table C in S1 File). Other 8 markers flanking *BADH2* in an interval of 59 cM were used for minimizing linkage drag. In the 210 lines of F7 generation, we obtained 8 lines which show similar agronomic phenotype to that of Jiachang1 while carrying the donor segment introgression of the *BADH2* gene. Among them, line 1446 was identified to be homozygous for the *BADH2* gene introgression, including an approximately 20.75 cM fragment between marker Os17800476 and Os22293835 (Fig 5).

## Discussion

The MAS strategy greatly accelerates the process of crop breeding by integrating molecular genetics with artificial selection [9]. However, a major limitation for applying this method is that recombination between markers and targeted genes would reduce the linkage disequilibrium, which could diminish the effectiveness of selection [64]. The development of new sequencing technologies narrows this gap because these technologies provide genome-wide sequence data for reliable marker identification with relatively low cost [65, 66]. With the increasing data generated from high-throughput sequencing platforms, effective data analysis is essential to further applications. A series of tools have been developed, which however could only support specific parts of a complete NGS data analysis [47, 67]. Therefore, it requires a combination of different technical resources with the additional bioinformatics expertise to finish the complex data analysis, which is cumbersome, especially for researchers with limited bioinformatics skill.

In this article, we integrated softwares including BWA, SAMtools, GATK and HeteroSNPPropLevel, to develop a software named AMF for RAD-seq data analysis. This software provides a graphical user interface and streamlined framework which covers quality assessment, filtering, mapping, variant detection, annotation and variant location. Each function is independent for a flexible application and can be manipulated easily with optional parameters to meet various needs. In addition to the integration of external tools, AMF provides three self-developed modules: SNP InDel Detection and Annotation, Somatic Detection and Variant Location, all of which are customizable for researchers to complete data mining. The output files of these three modules are either in TXT format or Excel spreadsheet format that are easy to understand and manipulate especially for those with minimal bioinformatics expertise. Further, unlike other integrated softwares, such as MAQ that provides command line interfaces [68], AMF provides users a visual and straightforward way to perform data analysis through a user-friendly graphical display, which greatly simplifies the analysis procedure. Additionally, AMF allows users to integrate other genomic information with high flexibility.

Based on this analysis platform, high-density rice markers were successfully identified from RAD tags with 93% accuracy, which could have been underestimated since PCR bias [69, 70], PCR errors, and sequencing errors [71] could all affect the final accuracy. To increase the accuracy, users could apply stricter parameters according to different NGS technologies and genomic features. In addition, repeated trials, and increased sequencing depth, and number of pooled samples may help reduce the error rate.

In addition to the identification of genome-wide markers, this study also demonstrated an application of AMF in MAS. In Variant Location module, the positions or regions of DNA makers could be easily located, which would assist users to determine the tightly linked markers for minimizing linkage drag, as exampled in the targeted breeding of *BADH2* (Fig 5). As more and more important genes or quantitative trait loci (QTLs) affecting growth and development of cereal crops are being cloned [72], genomics-assisted breeding will gain significant improvements for precise prediction of phenotypes from genotypes.

Development of AMF is an ongoing process. In current version, AMF only supports a Linux-based application. Efforts will be paid to extend it to support additional platforms for a wider use. Furthermore, because most of the polymorphic markers detected are SNPs, we have to further consider how to apply these markers at a lower cost. AMF will be continuously updated and extended according to the feedback provided by users. In conclusion, AMF provides an efficient strategy for large-scale and accurate marker discovery that can be widely used for basic research and MAS.

## Supporting Information

S1 File Statistics of the sequenced sites in JP69 and Jiaoyuan5A (Figure A). Marker validation (Figure B). Rice variety identification (Figure C). List of 117 important rice agronomic trait genes (Table A). Primer sequences used for accuracy verification and variety identification (Table B). Primer sequences used for target breeding of *BADH2* (Table C). Summary of sequence data (Table D).
(DOCX)
