# Supplementary material for: Development of a RAD-Seq Based DNA Polymorphism Identification Software, AgroMarker Finder, and Its Application in Rice Marker-Assisted Breeding
Source: PLoS One. 2016 Jan 22;11(1):e0147187. doi: 10.1371/journal.pone.0147187 (PMC4723255; doi:10.1371/journal.pone.0147187)
Supplement: S1 File — Statistics of the sequenced sites in JP69 and Jiaoyuan5A (Figure A). Marker validation (Figure B). Rice variety identification (Figure C). List of 117 important rice agronomic trait genes (Table A). Primer sequences used for accuracy verification and variety identification (Table B). Primer sequences used for target breeding of BADH2 (Table C). Summary of sequence data (Table D). (DOCX) [file pone.0147187.s001.docx]

**Supporting Information**


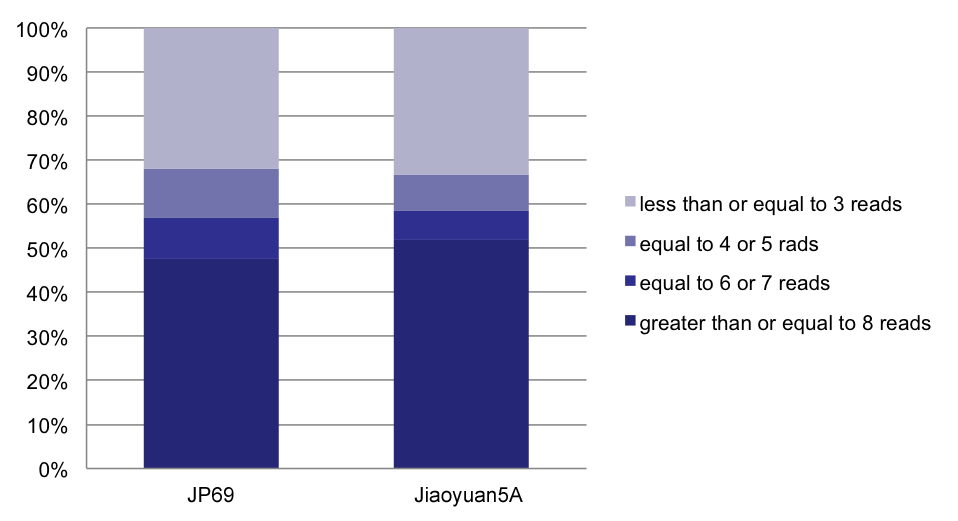


**Figure A. Statistics of the sequenced sites in JP69 and Jiaoyuan5A.**

The X axis shows two sequenced rice varieties; The Y axis indicates percentage of coverage.


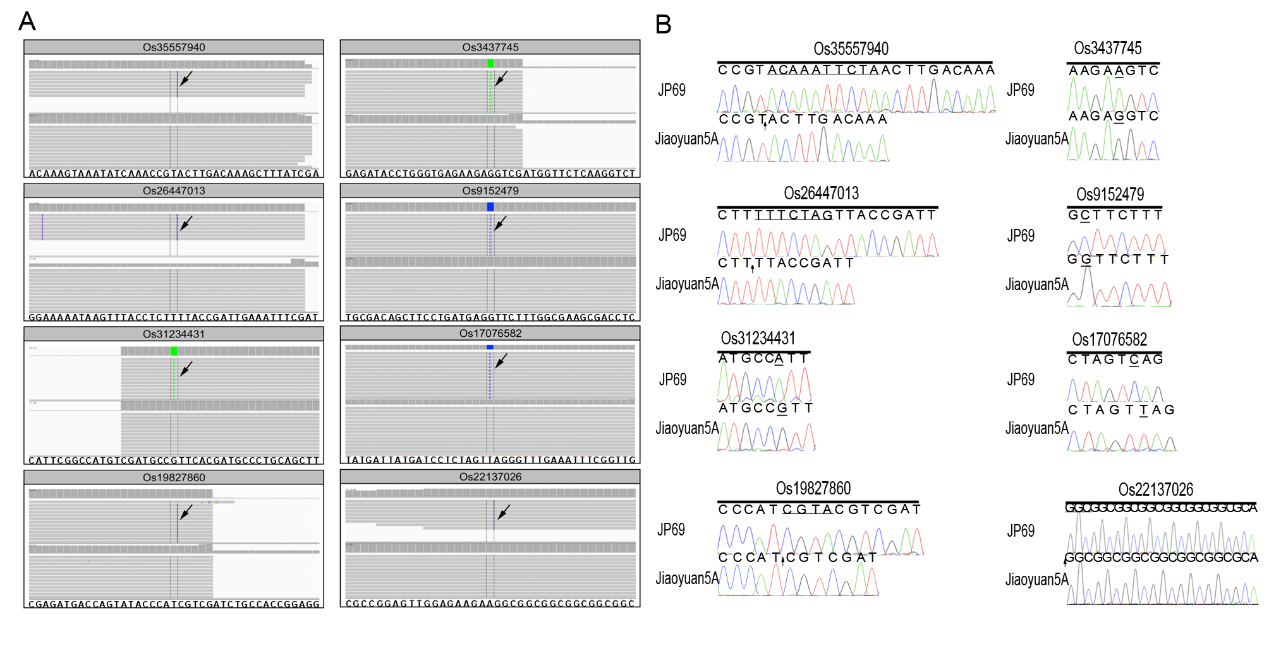
**Figure B. Marker validation.**

A, Integrative genomics viewer (IGV) shows alignment results of markers between JP69 and Jiaoyuan5A. JP69 is always showed in front. Arrows indicate the positions of polymorphic markers. B, Sanger sequencing validated the accuracy of markers. Among them, Os35557940, Os26447013, Os19827860 and Os22137026 are InDelswhileOs31234431, Os3437745, Os9152479 and Os17076582 are SNPs.

**
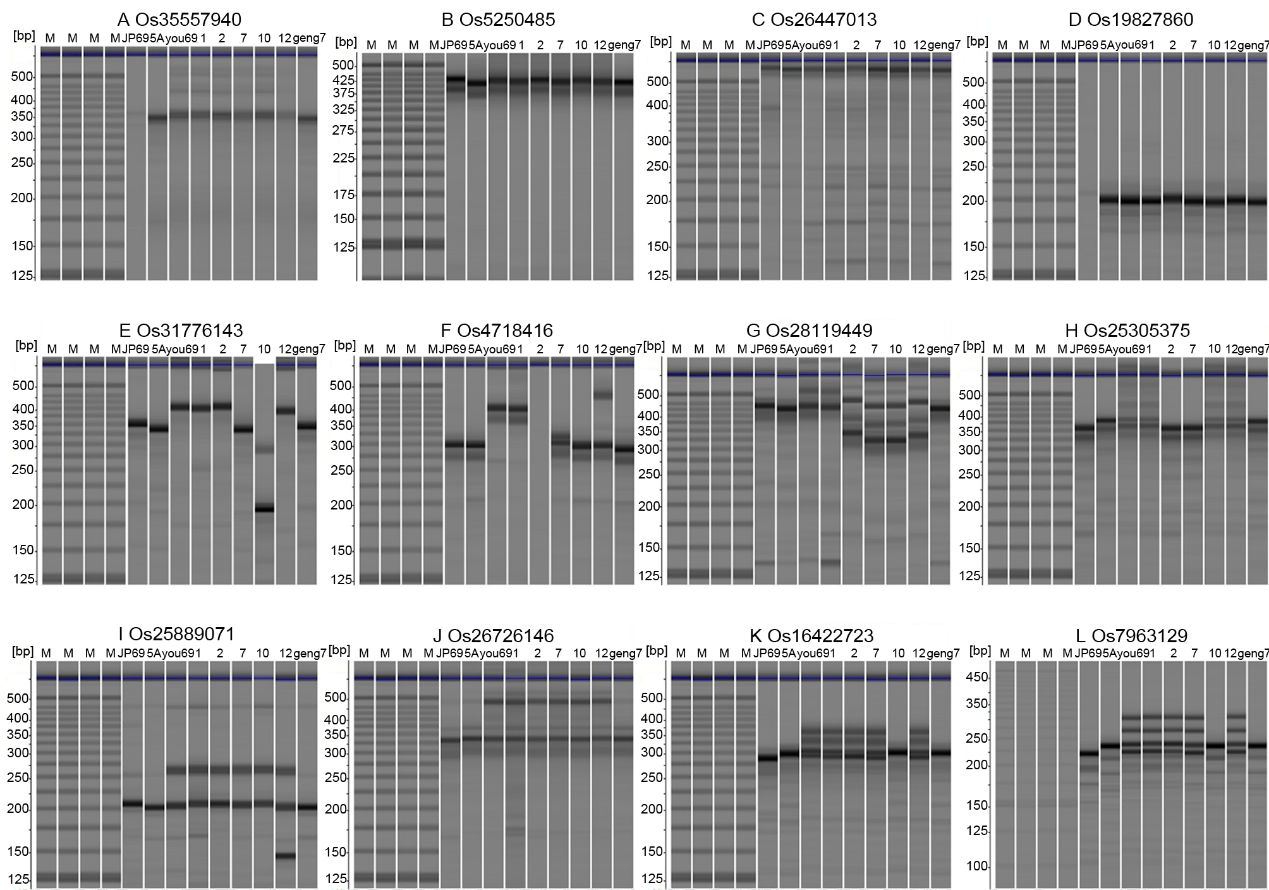
**

**Figure C. Rice variety identification.**

PCR products were run by the microchip electrophoresis system. In each picture, the first four lanes were markers and lanes5-13 are corresponding to JP69, Jiaoyuan5A (5A for short), Jiaoyuanyou69 (you69 for short), blind sample 1, blind sample 2, blind sample 7, blind sample 10, blind sample 12 and Wuyungeng7 (geng7 for short). A, Genomic DNA sequences were amplified by Os35557940F/Os35557940R, indicating that varieties 1, 2, 7, 10, 12 have similar amplified bands with Jiaoyuanyou69. B, Genomic DNA sequences were amplified by Os5250485F/Os5250485R, showing that varieties 1, 2, 7, 10, 12 have similar amplified bands with Jiaoyuanyou69. C, DNA sequences were amplified by Os26447013F/Os26447013R, indicating that varieties 1, 7, 10 have similar amplified bands with Jiaoyuanyou69. D, DNA sequences were amplified by Os19827860F/Os19827860R, showing that varieties 1, 7, 10, 12 have similar amplified bands with Jiaoyuanyou69. E, DNA sequences were amplified by Os31776143F/Os31776143R, indicating that varieties 1, 2 have similar amplified bands with Jiaoyuanyou69. F, Genomic DNA sequences were amplified by Os4718416F/Os4718416R, showing that only variety 1 has similar amplified band with Jiaoyuanyou69. G, Genomic DNA sequences were amplified by Os28119449F/Os28119449R, indicating that varieties 1, 7, 10 have similar amplified bands with Jiaoyuanyou69. H, DNA sequences were amplified by Os25305375F/Os25305375R, showing that varieties 1, 10, 12 have similar amplified bands with Jiaoyuanyou69. I, Genomic DNA sequences were amplified by Os25889071F/Os25889071R, indicating that varieties 1, 2, 7, 10 have similar amplified bands with Jiaoyuanyou69. J, DNA sequences were amplified by Os26726146F/Os26726146R, showing that varieties 1, 2, 7, 10, 12 have similar amplified bands with Jiaoyuanyou69. K, Genomic DNA sequences were amplified by Os16422723F/Os16422723R, indicating that varieties 1, 2, 7, 12 have similar amplified bands with Jiaoyuanyou69. L, DNA sequences were amplified by Os7963129F/Os7963129R, showing that varieties 1, 2, 7, 12 have similar amplified bands with Jiaoyuanyou69.

**Table A. List of 117 important rice agronomic trait genes.**

| **Traits** | **QTL/Gene** | **RAP-ID** | **Marker** | **Chr** | **Type** | **InDel Length** | **JP_seq** | **Jiaoyuan5A_seq** | **Forward primer(5'-3')** | **Reverse primer(5'-3')** | **References** |
| --- | --- | --- | --- | --- | --- | --- | --- | --- | --- | --- | --- |
| disease resistance related | Pi-t | Os01g0149500 | Os2683065 | 1 | SNP |  | A | C | TGAGTACAAATGAACCCCCT | ATCATACCAGAGTCAGTGGG | Kiyosawa et al., 1972; Hayashi et al., 2010 |
|  | SKC1 | Os01g0307500 | Os11461717 | 1 | SNP |  | C | T | AATCAAGGGCTCCAACAAACT | CTCTTCATGGCGGTCAACTC | Lin et al., 2004; Cotsaftis et al., 2012 |
|  | OsKAT1 | Os01g0756700 | Os31756812 | 1 | SNP |  | T | C | CGACGACCATAAGCGAGTT | TAATGGCTCACCTCGTTTTG | Obata et al., 2007 |
|  | OsCIPK12 | Os01g0759400 | Os31942545 | 1 | SNP |  | T | C | TGTTCATGTAGGCATTAGCG | AAAGCCAAACGAGAAACACT | Xiang et al., 2007 |
|  | Pish | Os01g0782100 | Os33170071 | 1 | SNP |  | T | C | CCCCGTACTGGTAGTTGTC | CTCCTGGTGTCGTTCCTGTT | IMBE et al., 1985; Takahashi et al., 2010 |
|  | OsMYB3R-2 | Os01g0841500 | Os36129346 | 1 | SNP |  | C | T | GTGGATGCCCTTAGGAAAAG | GATTGTGAGACGACTAGTGC | Dai et al., 2007; Ma et al., 2009 |
|  | OsNAC6/SNAC2 | Os01g0884300 | Os38398054 | 1 | SNP |  | T | G | AAAGTTCGAGGAAAGTGACG | GAAGCTAGCCATGGTTTTCA | Ohnishi et al., 2005; Nakashima et al., 2007 |
|  | OsLOX1 | Os03g0700700 | Os5250485 | 2 | InDel | 14 | AATGTGAACCCAGAT | A | CCATCAGACTTTTCGACAGG | CAGAATATTACCCCCTCCGT | Wang et al., 2008 |
|  | AP59 | Os02g0654700 | Os26447013 | 2 | InDel | 7 | TTTTCTAG | T | GAGCCGCTGATGATTGGT | AAAGCCGGTAGATTCCAAAC | Oh et al., 2009 |
|  | OsSKIPa | Os02g0759800 | Os31993051 | 2 | InDel | 1 | C | CG | GATCGACTGATTCTACAGCG | AGGCTTCCTTTGATTCGAAC | Hou et al., 2009 |
|  | OsbZIP23 | Os02g0766700 | Os32275394 | 2 | SNP |  | C | T | CTCATGCTCATTGCTCTCAA | ACCCCAACCAAACAATTCTC | Xiang et al., 2008; Nijhawan et al., 2008 |
|  | DSM2 | Os03g0125100 | Os1241431 | 3 | SNP |  | T | C | GCATTGATAGGATCGCACTT | GTCGGGTTTTCCAAGTCTTT | Du et al., 2010 |
|  | Bph14/Qbp1 | Os03g0848700 | Os35693885 | 3 | InDel | 1 | C | CT | CCTCTGATTCTGGCAAACAA | GCGAGGATGACTTGACATAC | Huang et al., 2001; Du et al., 2009 |
|  | pi-21 | Os04g0401000 | Os19827860 | 4 | InDel | 4 | TCGTA | T | CACTTACCTCTCAGGCTACA | TACCTTTCCTCTTCGTCTCC | Fukuoka et al., 2001; Fukuoka et al., 2009 |
|  | xa-5 | Os05g0107700 | Os437683 | 5 | SNP |  | A | C | GCCATTCAAGTTCTTGTCCA | TGAGTAGGCTCGAAACAGAT | Blair et al., 1997; Iyer et al., 2004; Iyer-Pascuzzi et al., 2008 |
|  | OsSIK1 | Os06g0130100 | Os1524202 | 6 | SNP |  | G | A | TTCCTGCTGTTCTTGTTCAG | GAATTGCATTGGTGATCAGC | Qiang et al., 2010 |
|  | Pi9/Pi2/Piz-5/Pi50/Piz/Pigm/Piz-t | Os06g0286700 | Os10385438 | 6 | SNP |  | C | T | AGTGGACACTGGTCTATCTG | AAAGAGAAGCCGAACACATC | Zhuang et al., 1998; Chen et al., 2001; Jiang et al., 2012 |
|  | Pi25/Pid3 | Os06g0330100 | Os12929694 | 6 | SNP |  | A | G | TGTTTCCAATTTCGCGTACT | ATGCATGCCAAACAAAAGTG | Zhuang et al., 1997; Zhuang et al., 1998; Chen et al.,2011 |
|  | Pid2 | Os06g0494100 | Os17181894 | 6 | SNP |  | A | G | GTTAGTGCCGCTAAGTTGAA | CGACGGTAATATTTCCCGAC | Chen et al., 2004; Chen et al., 2010; Kouzai et al., 2013 |
|  | OsCIPK03 | Os07g0687000 | Os29190825 | 7 | SNP |  | A | G | GATTGAACATGGCTCTGTGT | TAAGCTAATCTGCAAGGTGC | Xiang et al., 2007 |
|  | OsHI-LOX | Os08g0508800 | Os25216595 | 8 | SNP |  | G | A | GCAGATAAGAATGGTGCCAA | AGACAAAACGCTCCAAAACT | Zhou et al., 2009 |
|  | xa13/Os-8N3 | Os08g0535200 | Os26726146 | 8 | InDel | 4 | G | GCTGA | GAGATTGCAACACGGTAAGT | CTGATGAAGAAGACGACGAC | Ogawa et al., 1987; Antony et al., 2010; Yuan et al., 2011 |
|  | Pi5/Pi3/Pi-i | Os09g0327600 | Os9619154 | 9 | SNP |  | C | T | CAAATTTGGCTAGTGGAGGG | CACGTGGGTCCGATTTTATT | Wang et al., 1994; Yi et al., 2004; Lee et al., 2009 |
|  | Pi56(t)/qBR9.1 | Os09g0328951 | Os9802102 | 9 | SNP |  | T | C | CATGTCTTGGCTAGGATTCG | CGCTGACGTTGGGTATTTAT | Liu et al., 2011; Liu et al., 2013 |
|  | MYBS3 | Os10g0561400 | Os22137026 | 10 | InDel | 3 | AGGC | A | CGTCTCTTGCTCTATTTCGG | ATTTACTGGATCTCCCTCCC | Su et al., 2010 |
|  | OsCIPK15 | Os11g0113700 | Os576135 | 11 | InDel | 1 | GA | G | ATGAAAGAAAGAGCCGCAAT | ATATACCGTGCAATCAAGCA | Lee et al., 2009; Kudahettige et al., 2011 |
|  | OsNAC10 | Os11g0126900 | Os1229569 | 11 | SNP |  | C | T | GAGTACACCCTACATCCGAA | GGACAGTGTGGTAGAGAAGA | Jeong et al., 2010; Sun et al., 2013 |
|  | Pi-a/RGA4/RGA5 | Os11g0225100 | Os6532244 | 11 | SNP |  | C | A | CACCATGACAAGGATGCTTC | TCATCGTTTGGAAGCGTATC | Kiyosawa et al., 1972; Cesari et al., 2013 |
|  | OsGH3.13/TLD1 | Os11g0528700 | Os19179120 | 11 | SNP |  | G | A | TCCACATGCAAAGGTTTGAA | TCATGATTGCTACGACGAAC | Jain et al., 2006; Zhang et al., 2009 |
|  | Xa21 | Os11g0559200 | Os20801374 | 11 | InDel | 3 | C | CTCT | AGACACGTTATGACACCAAC | TTAAGCAAAAGTCGATGGCA | Ronald et al., 1992; Gan et al., 2011; Park et al., 2012 |
|  | Pik-h/Pi-54/Pi54rh | Os11g0639100 | Os25241514 | 11 | InDel | 3 | A | ACAC | TTTGAAGTTGCCATGGGTAG | TTCGTTTCTGATATGACCGC | Sharma et al., 2005; Gupta et al., 2012 |
|  | Pita/Pi-4a | Os12g0281300 | Os10606779 | 12 | SNP |  | G | A | GCACCAGGAATCTTGTCAAT | GTATCGAAGAAGACATGCCC | Inukai et al., 1996; Wang et al., 2010; Lee et al., 2011 |
|  | Xa25/x25 | Os12g0476200 | Os16583663 | 12 | SNP |  | G | C | GTCGTCCTCGTTTCTGTAGTAGTTG | TCGATTACGGAGTGGGTGGTG | Chen et al., 2002; Liu et al., 2011 |
| yield related | d18/OsGA3ox2 | Os01g0177400 | Os4002750 | 1 | InDel | 2 | C | CAT | CGACTCCCTCAAACAGAATC | GCCATGGATGTGTAAGCTAG | Itoh et al., 2001; Iwamoto et al., 2011 |
|  | d-2/CYP90D2 | Os01g0197100 | Os5234385 | 1 | SNP |  | A | C | CTCACTTAAGGCTCAGCTTG | AGACAAGTTGAGAGACCCAA | Hong et al., 2003 |
|  | Gn1a/OsCKX2 | Os01g0197700 | Os5269977 | 1 | SNP |  | C | G | GTCACAAATCTCTCCACGTC | CTATCGTCGTCTTGTGGG | Ashikari et al., 2005 |
|  | LOG | Os01g0588900 | Os22931446 | 1 | InDel |  | TA | T | CGTTTTTGTGAACAACGTGT | TTTGGCCTCCATTGATATGC | Kurakawa et al., 2007 |
|  | d61/OsBRI1 | Os01g0718300 | Os29929026 | 1 | SNP |  | G | A | GTAGACATCACCAAACCCAC | AGTTCCCCAAGATCTTCCAA | Yamamuro et al., 2000; Sakamoto et al., 2013 |
|  | D10 | Os01g0746400 | Os31225473 | 1 | SNP |  | G | T | TTCATGCTGCCATTTTCGTA | TGCCTCGTTTCGTTTGTAAT | Arite et al., 2007; Ito et al., 2010; Zhang et al., 2010 |
|  | LAX1 | Os01g0831000 | Os35557940 | 1 | InDel | 10 | TACAAATTCTA | T | TCACTGAACCACCAAATCAC | TGCATATATATACTGCGCGC | Komatsu et al., 2001; Oikawa et al., 2009 |
|  | sd1/GA20ox-2 | Os01g0883800 | Os38381393 | 1 | SNP |  | G | A | CATGGTCAGGCATAAACCAA | CTTAGAGCACGTGTAACACC | Sasaki et al.,2002; Spielmeyer et al., 2002 |
|  | GW2 | Os02g0244100 | Os8061228 | 2 | SNP |  | T | C | TCCAAACCCTAACCTCAACCAT | CGGCATGGTCAAAGTGGAAA | Song et al., 2007 |
|  | RPBF | Os02g0252400 | Os8596013 | 2 | SNP |  | A | G | AAACGCAATTCGAAAGATGC | CATCAACCCGCTGTTCATAA | Yamamoto et al., 2006; Kawakatsu et al., 2009 |
|  | EP3/LP | Os02g0260200 | Os9038393 | 2 | SNP |  | A | G | AGCTTGTTGTTCGAGAATCC | AGGGTTATTACCTGACCGTT | Piao et al., 2009 |
|  | RCN2 | Os02g0531600 | Os19568704 | 2 | SNP |  | A | G | CCAATGTGAAGAAGGACCTG | CAGAGAGAGAGCTGAGAGAG | Nakagawa et al., 2002 |
|  | GID2 | Os02g0580300 | Os22348468 | 2 | SNP |  | C | T | CTGGTGGGGTTTGGTTTTAA | AGGTTCTCACTCGAGGAAAA | Sasaki et al., 2003; Gomi et al., 2004 |
|  | DTH2 | Os02g0724000 | Os30097070 | 2 | InDel | 2 | C | CTT | GATGGGATGAATCTTCTGCC | AGGAAAGCTTTGACAGTGTG | Wu et al., 2013 |
|  | Ehd4 | Os03g0112700 | Os753135 | 3 | InDel | 16 | T | TCGTACAGGCAACGATG | GTTTTCGTCGTCGTCAAATT | AAACGAAGGCTAGCAACAAT | Gao et al., 2013 |
|  | TAD1/TE | Os03g0123300 | Os1241431 | 3 | SNP |  | T | C | GCATTGATAGGATCGCACTT | GTCGGGTTTTCCAAGTCTTT | Su’udi et al., 2012 |
|  | PGL1 | Os03g0171300 | Os3811749 | 3 | SNP |  | T | G | CTCAAAAGCTGAGCCACC | GACACCTGACATGGAGTAGA | Heang et al., 2012 |
|  | HTD2/D88/D14 | Os03g0203200 | Os5425050 | 3 | SNP |  | A | G | CATTCCTACATCAGCCATGG | TCAACGATTTTGGGCTTGAG | Liu et al., 2009; Arite et al., 2009 |
|  | OsDWARF4/CYP90B2 | Os03g0227700 | Os6738491 | 3 | SNP |  | T | C | CATCTCCCTCAACTTCCTCT | ATCAGGTTGAACGTGAACTG | Sakamoto et al., 2006 |
|  | GS3 | Os03g0407400 | Os16729065 | 3 | InDel | 1 | GC | G | CAACCACCATATCAGCCAAA | CCTTGCTCTTAGTAATGGCC | Fan et al., 2006; Takano-Kai et al., 2011 |
|  | OsTB1/FC1 | Os03g0706500 | Os28396291 | 3 | SNP |  | A | C | AGCCATGCAAATGTTCTTCT | AGCACAATGTTGGTATGGAC | Takeda et al., 2003; Xia et al., 2012 |
|  | PAP2 | Os03g0753100 | Os31048249 | 3 | InDel | 2 | T | TAC | TTTCATACCTTGCACGTGAG | AGTACTGTCACTGCGAGTAA | Gao et al., 2010; Kobayashi et al., 2010 |
|  | Nglf-1 (OsARG) | Os04g0106300 | Os396921 | 4 | SNP |  | A | T | TCAAGGTCCTCTCTCTCTCT | GAGAGAGAGAGAGCAAGTGT | Ma et al., 2013 |
|  | LAX2 | Os04g0396500 | Os19562176 | 4 | SNP |  | T | C | ATGGGAGAAAAAGCGAAGAC | GGCACAAAGGACAACAAAAG | Tabuchi et al., 2011 |
|  | d11/CYP724B1 | Os04g0469800 | Os23487855 | 4 | SNP |  | A | G | GCAACTAGCTAGAGCCAATC | TACAACCAGGATCCAAAGGA | Tanabe et al., 2006 |
|  | D17/HTD1 | Os04g0550600 | Os27564581 | 4 | InDel | 1 | A | AT | GGTGCCTCTCTTCTCATACT | CATGCCCATGTTATTCCCAA | Zou et al., 2005 |
|  | APO2/RFL | Os04g0598300 | Os30182639 | 4 | SNP |  | A | T | TTGGTCAGGTGGATTTCTT | TGTAAGGCAGTTAGGTCGTT | Rao et al., 2008; Ikeda‐Kawakatsu et al., 2012 |
|  | FLO2 | Os04g0645100 | Os32834729 | 4 | SNP |  | G | C | AGAAGGAGAGAATAGTGGCC | ACTCTGGCTTGGTAATGACT | Kaushik et al., 1991; She et al., 2010 |
|  | SRS3 | Os05g0154700 | Os3210838 | 5 | SNP |  | A | C | GCATTAGTTCGTTCGTCCCT | GAACACATAGATTCCCACCG | Tanabe et al., 2007; Kitagawa et al., 2010 |
|  | GS5 | Os05g0158500 | Os3437745 | 5 | SNP |  | A | G | GCTCTTGAAAATCTCGGTGT | GTCTCCTTGCATGTACCTTC | Yu et al., 1997; Xing et al., 2002 |
|  | qSW5/GW5 | Os05g0187500 | Os5360210 | 5 | SNP |  | T | C | AACCAACGCCGATGTTATAC | GAACAACATCGATGCGTCTA | Wan et al., 2005; Weng et al., 2008 |
|  | D1/RGA1 | Os05g0333200 | Os15598506 | 5 | SNP |  | G | A | TATGCGTTCTGTAATCCCCT | CTGCATAGAAAGGGTACGTG | Ishikawa et al., 1995; Wang et al., 2006; Miura et al., 2009 |
|  | GID1 | Os05g0407500 | Os19869877 | 5 | SNP |  | A | G | AGCTGTCGTACAACATTCTG | CTGGAGCGTCACGAAGTA | Ueguchi-Tanaka et al., 2005; Yamamoto et al., 2010 |
|  | Eui | Os05g0482400 | Os23742196 | 5 | SNP |  | C | T | GAGTTTGTCATCTACCGGTG | GGGATGACATGTATGAGCAC | Yang et al., 2002; Zhu et al., 2006 |
|  | DLT | Os06g0127800 | Os1524202 | 6 | SNP |  | G | A | TTCCTGCTGTTCTTGTTCAG | GAATTGCATTGGTGATCAGC | Tong et al., 2009; Li et al., 2010; Tong et al., 2012 |
|  | D3 | Os06g0154200 | Os2773379 | 6 | SNP |  | G | A | CCACTGTATGCAGATTTCCC | GCGCTTAATTTGTGATGTGG | Yan et al., 2007 |
|  | HGW | Os06g0160400 | Os3432838 | 6 | SNP |  | T | G | TAGGAGACGAGGAACCTAGT | TACTTTGCTTTTCGCCTGAA | Li et al., 2012 |
|  | Hd1 | Os06g0275000 | Os9332768 | 6 | SNP |  | A | G | CAGTCGTCCCATTTGTTCAT | TAAGCAGATTAGCGGCAAAG | Yano et al., 1997; Yamamoto et al., 1998; Huang et al., 2012 |
|  | MOC1 | Os06g0610350 | Os24340417 | 6 | InDel | 9 | TATAAGCCAG | T | GGAATACGTAGGCATGCAAT | TGCATTCGTGCTTAGAACAA | Li et al., 2003; Lu et al., 2009 |
|  | TGW6 | Os06g0623700 | Os25095178 | 6 | SNP |  | C | T | AATCACGCAGCAGATCAATT | AAACCACTTCACAACTCCAC | Ishimaru et al., 2013 |
|  | OsPIN2 | Os06g0660200 | Os27219152 | 6 | SNP |  | T | C | TGCCCATAGTAGTTTCTCGT | AATTAGTAGAGGGCTCGAGG | Chen et al., 2012 |
|  | SCM2/APO1 | Os06g0665400 | Os27480778 | 6 | SNP |  | C | G | AACAACAACCAAGGCAAGAT | CTCGCCAACCCGATCAC | Ikeda et al., 2007; Ookawa et al., 2010 |
|  | DEP3 | Os06g0677000 | Os28119449 | 6 | InDel | 6 | AAAAAAC | A | CACCGTTTGTTCATCTCCAT | CTTAATCGCTCTGTGATGCA | Qiao et al., 2011 |
|  | d6/OSH15/Oskn3 | Os07g0129700 | Os1560741 | 7 | SNP |  | T | C | ATCAATTCGAAGTGGACGTG | ATCCACTGTAACCTACGGTT | Sato et al., 1998; Sentoku et al., 1999; Ito et al., 2008 |
|  | PROG1 | Os07g0153600 | Os2832225 | 7 | InDel | 2 | A | AAG | GTGTCGCTTCAGATCATCTC | GGACACGAAAACGCATTACT | Wang et al., 2008; Jin et al., 2008 |
|  | RISBZ1 | Os07g0182000 | Os4322489 | 7 | SNP |  | C | T | CTCAGCAAGTCCAAAATCCA | TGTGGATTCTGTCGAGAAGA | Onodera et al., 2001; Yamamoto et al., 2006 |
|  | Ghd7 | Os07g0261200 | Os9152479 | 7 | SNP |  | C | G | ATCTGAACCATTGTCCAAGC | CATATTGTGGGAGCACGTTC | Xue et al., 2008; Shibaya et al., 2011; Weng et al., 2014 |
|  | EP2/DEP2/SRS1 | Os07g0616000 | Os25382707 | 7 | SNP |  | C | T | GGTGGAATGATCTTGCAGTT | ATAACTTGTGAAGGCACTCG | Tanabe et al., 2007; Abe et al., 2010 |
|  | FZP | Os07g0669500 | Os28252688 | 7 | SNP |  | T | A | CTGAACCACGTGATGAGTTT | ATCTGAACTGAAGCTTGCAG | Komatsu et al., 2003; Kato et al., 2012 |
|  | ASP1 | Os08g0162100 | Os3660969 | 8 | SNP |  | T | C | CACCAATGAATGAGGGTGTT | ATTCTCCATGCCACATGTTC | Gao et al., 2012; Kwon et al., 2012; Yoshida et al., 2012 |
|  | Ghd8/DTH8 | Os08g0174500 | Os4570420 | 8 | SNP |  | T | C | TACCATCGGTGAATGCTAGT | CTGAACCCTTCTAGCAGGTA | Zhang et al., 2006; Thirumurugan et al., 2008; Dai et al., 2012 |
|  | OsSPL14/IPA1/WFP | Os08g0509600 | Os25305375 | 8 | InDel | 9 | G | GTATGTACTA | CATATCATCCAGCACAAGCA | GCTAGATTGGCCGGTTAAAT | Springer et al., 2010; Miura et al., 2010 |
|  | qGW8/OsSPL16 | Os08g0531600 | Os26508206 | 8 | SNP |  | G | A | CTCATCATCTTCTCCAACCG | CAGATGCAGCTAGCTTTTGA | Wang et al., 2012 |
|  | OsSPY | Os08g0559300 | Os27991672 | 8 | SNP |  | C | T | TGCTCCCACAAGGAAAGT | GTAGGAGGAGAGCTCACATT | Shimada et al., 2006; Phanchaisri et al., 2012 |
|  | DEP1/DN1/qPE9-1 | Os09g0441900 | Os16422723 | 9 | InDel | 12 | A | AACCTTCATGTCC | TCTGTTCCCCACCTCTATTC | TGGATGACTTCTTCCCAGTT | Yan et al., 2007; Zhou et al., 2009; Taguchi-Shiobara et al., 2011 |
|  | SG1 | Os09g0459200 | Os17348611 | 9 | SNP |  | A | T | CCAACTCATTGTCGACTTGT | CTTGTTGTCGCTAGATCCTG | Nakagawa et al., 2012 |
|  | Ehd1 | Os10g0463400 | Os17076582 | 10 | SNP |  | C | T | TGCTTGTTGTTGGAATCGAT | GGGTGAACGAAATTACCGAA | Doi et al., 1998; Endo-Higashi et al., 2011 |
|  | RCN1 | Os11g0152500 | Os2450044 | 11 | SNP |  | C | T | CGTCGATCACTTAGGGAAAC | CTAGCTCGCTGGATCTTCA | Nakagawa et al., 2002 |
|  | SP1 | Os11g0235200 | Os7205693 | 11 | SNP |  | C | T | TGCCTGGTCTTCTCTGTTAA | TGTCTAATGAAAAGCGTGCA | Li et al., 2009 |
|  | SRS5 | Os11g0247300 | Os7963129 | 11 | InDel | 16 | G | GTCCGTGTGGGCTTCTA | GGTGACGAGTACTAGAGAGG | AGATGCCACAAAGAAGGTTC | Segami et al., 2012 |
|  | D27 | Os11g0587000 | Os22222234 | 11 | InDel | 1 | A | AT | TGCCATGGCTGATTTGATTT | CGGAGAACACATCACATCTC | Lin et al., 2009 |
| rice quality | AGPiso | Os01g0633100 | Os25353076 | 1 | SNP |  | G | A | GATAATGCCCCCATAAGCTG | AAACAGTGTAATTGAGCCCC | Jeon et al., 2010 |
|  | SSIV-1 | Os01g0720600 | Os30027392 | 1 | SNP |  | C | T | TGCCTGCCTCTTTTGATTTT | GGAGTAGGCTTCTCTCACAT | Hirose et al., 2004 |
|  | qSH1 | Os01g0848400 | Os36460075 | 1 | SNP |  | T | C | TCTCTCTCAAAAAGTCCGGT | ATTGAAGGGAGTAGCAACCT | Konishi et al., 2006; Zhang et al., 2009 |
|  | SBE3 | Os02g0528200 | Os19355345 | 2 | SNP |  | A | T | CAGTCATAGCAGCTCATGTC | TCCTCTCCGAGATGTTAACC | Tian et al., 2009 |
|  | OsbZIP22 | Os02g0728001 | Os30316456 | 2 | InDel | 1 | AT | A | CTATGCCTAGGAAGTGCTCT | AGTTCAAAGTGCCTGAGTTC | Nijhawan et al., 2008; Ji et al., 2009 |
|  | SSII-2 | Os02g0744700 | Os31234431 | 2 | SNP |  | A | G | CCTCCTTGCACTGTTTCTTG | CATTTCAGACTGTACGACCC | Hirose et al., 2004 |
|  | PUL | Os04g0164900 | Os4396588 | 4 | SNP |  | G | A | CGGCATTGAAGTACTCGATG | CCTGCCTCTTACAACTTCAC | Fujita et al., 2009 |
|  | SBE4 | Os04g0409200 | Os20231704 | 4 | SNP |  | G | A | GGAAGATGAGTCACTCCTCA | TAAGTTGTCAGCACATGTGG | Regina et al., 2005 |
|  | GIF1/OsCIN2 | Os04g0413500 | Os20421470 | 4 | InDel | 1 | GA | G | GTGGAATAAGCTAAACGGCA | ACAGGCCATTACGAAAATGT | Wang et al., 2008; Wang et al., 2010 |
|  | SSIII-1 | Os04g0624600 | Os31776143 | 4 | InDel | 8 | TCTCCTATC | T | GTGTAAAGGGGAGCGATTTT | GGGACCCAAAAACCTTGAAT | Hirose et al., 2004; Fujita et al., 2007 |
|  | SSIV-2 | Os05g0533600 | Os26480678 | 5 | SNP |  | G | A | TCCTCATCCTTCTCAACCTC | CTCCATCTCCTTCCACCTC | Hirose et al., 2004 |
|  | wx/qGC-6/Wx-mq/Wx-op | Os06g0133000 | Os762888 | 6 | SNP |  | A | G | CCTTCATCTATGGCCGATTG | GAGATATGCCTAGGAGGACC | Sano et al., 1984; Isshiki et al., 1998; Zhang et al., 2012 |
|  | SSI | Os06g0160700 | Os3432838 | 6 | SNP |  | T | G | TAGGAGACGAGGAACCTAGT | TACTTTGCTTTTCGCCTGAA | Fujita et al., 2006 |
|  | PTF1 | Os06g0193400 | Os4718416 | 6 | InDel | 4 | TCTGG | T | TGGGTTACCGTAGCACTTAT | TAATTAGCGCGGCGAAATTA | Yi et al., 2005 |
|  | ALK | Os06g0229800 | Os6747948 | 6 | SNP |  | C | G | CGACTATGGTTTGTGTGTGT | CGGAGGAATAGGTGGTTTTG | He et al., 1999; Kawakatsu et al., 2009 |
|  | SBE1 | Os06g0726400 | Os30892084 | 6 | SNP |  | G | T | GATAACCACTGATCGGATGC | ACGAAATGCAAGTCTCTGTC | Satoh et al., 2003 |
|  | GBSSII | Os07g0412100 | Os12918436 | 7 | SNP |  | T | C | CTTGCAATTGGATGAGACCA | AAGCAAGGGCACTCAATAAG | Dian et al., 2003 |
|  | DPE2 | Os07g0662900 | Os28252688 | 7 | SNP |  | T | A | TTGGCTGGACCATCATTCG | CTTTCAGTCTCAGGCGACCAG | Lu et al., 2004 |
|  | SSIII-2 | Os08g0191433 | Os5342240 | 8 | SNP |  | G | A | CTCAAGCTTGAAGGCATCAT | ATTAAACCATTGCCCCAGAC | Fujita et al., 2007 |
|  | BADH2 | Os08g0424500 | Os20367945 | 8 | SNP |  | C | T | CATCATCTCCACCATCACAC | CCTACGTGGCAGTCTAGTTA | Chen et al., 2006; Kuaprasert et al., 2011 |
|  | ISA | Os08g0520900 | Os25889071 | 8 | InDel | 4 | CTAAT | C | GGGGCAAATGAACAGCTC | ATAAATCTCCCGCCATTTCG | Fujita et al., 1999; Tian 2009 |
|  | AGPsma | Os09g0298200 | Os7234952 | 9 | InDel | 2 | A | ACT | CGAGGAACACAATCTCGATC | ATGACCATACGGAGGCAC | Bao et al., 2013 |
|  | SSII-1 | Os10g0437600 | Os15672325 | 10 | SNP |  | C | T | TTGGATATGTTGACGTCTGC | TCAGATGGTCATCGACAAGA | Hirose et al., 2004 |

**Table B. Primer sequences used for accuracy verification and variety identification.**

| **Primer name** | **Sequence (5’ – 3’)** | **Purpose** |
| --- | --- | --- |
| Os2683065F | TGAGTACAAATGAACCCCCT | accuracy verification |
| Os2683065R | ATCATACCAGAGTCAGTGGG | accuracy verification |
| Os4002750F | CGACTCCCTCAAACAGAATC | accuracy verification |
| Os4002750R | GCCATGGATGTGTAAGCTAG | accuracy verification |
| Os5233163F | GTCCAGTCAGCGTTCTATTC | accuracy verification |
| Os5233163R | CAGAGGTGGAGGTGTCAGT | accuracy verification |
| Os5234385F | CTCACTTAAGGCTCAGCTTG | accuracy verification |
| Os5234385R | AGACAAGTTGAGAGACCCAA | accuracy verification |
| Os5269977F | GTCACAAATCTCTCCACGTC | accuracy verification |
| Os5269977R | CTATCGTCGTCTTGTGGG | accuracy verification |
| Os11461717F | AATCAAGGGCTCCAACAAACT | accuracy verification |
| Os11461717R | CTCTTCATGGCGGTCAACTC | accuracy verification |
| Os22931446F | CGTTTTTGTGAACAACGTGT | accuracy verification |
| Os22931446R | TTTGGCCTCCATTGATATGC | accuracy verification |
| Os25353076F | GATAATGCCCCCATAAGCTG | accuracy verification |
| Os25353076R | AAACAGTGTAATTGAGCCCC | accuracy verification |
| Os29929026F | GTAGACATCACCAAACCCAC | accuracy verification |
| Os29929026R | AGTTCCCCAAGATCTTCCAA | accuracy verification |
| Os30027392F | TGCCTGCCTCTTTTGATTTT | accuracy verification |
| Os30027392R | GGAGTAGGCTTCTCTCACAT | accuracy verification |
| Os31225473F | TTCATGCTGCCATTTTCGTA | accuracy verification |
| Os31225473R | TGCCTCGTTTCGTTTGTAAT | accuracy verification |
| Os31756812F | CGACGACCATAAGCGAGTT | accuracy verification |
| Os31756812R | TAATGGCTCACCTCGTTTTG | accuracy verification |
| Os31942545F | TGTTCATGTAGGCATTAGCG | accuracy verification |
| Os31942545R | AAAGCCAAACGAGAAACACT | accuracy verification |
| Os33170071F | CCCCGTACTGGTAGTTGTC | accuracy verification |
| Os33170071R | CTCCTGGTGTCGTTCCTGTT | accuracy verification |
| Os35557940F | TCACTGAACCACCAAATCAC | accuracy verification, variety identification |
| Os35557940R | TGCATATATATACTGCGCGC | accuracy verification, variety identification |
| Os36120716F | ATTGCAAAAGCTTGTGCATC | accuracy verification |
| Os36120716R | CAATGATCCTTATCCCTGCC | accuracy verification |
| Os36129346F | GTGGATGCCCTTAGGAAAAG | accuracy verification |
| Os36129346R | GATTGTGAGACGACTAGTGC | accuracy verification |
| Os36460075F | TCTCTCTCAAAAAGTCCGGT | accuracy verification |
| Os36460075R | ATTGAAGGGAGTAGCAACCT | accuracy verification |
| Os38381393F | CATGGTCAGGCATAAACCAA | accuracy verification |
| Os38381393R | CTTAGAGCACGTGTAACACC | accuracy verification |
| Os38398054F | AAAGTTCGAGGAAAGTGACG | accuracy verification |
| Os38398054R | GAAGCTAGCCATGGTTTTCA | accuracy verification |
| Os5250485F | CCATCAGACTTTTCGACAGG | accuracy verification, variety identification |
| Os5250485R | CAGAATATTACCCCCTCCGT | accuracy verification, variety identification |
| Os8150174F | CGACTTCAGGGATAAGCATG | accuracy verification |
| Os8150174R | GTATACGCGAGACTGAGGTA | accuracy verification |
| Os8061228F | TCCAAACCCTAACCTCAACCAT | accuracy verification |
| Os8061228R | CGGCATGGTCAAAGTGGAAA | accuracy verification |
| Os8596013F | AAACGCAATTCGAAAGATGC | accuracy verification |
| Os8596013R | CATCAACCCGCTGTTCATAA | accuracy verification |
| Os9038393F | AGCTTGTTGTTCGAGAATCC | accuracy verification |
| Os9038393R | AGGGTTATTACCTGACCGTT | accuracy verification |
| Os19355345F | CAGTCATAGCAGCTCATGTC | accuracy verification |
| Os19355345R | TCCTCTCCGAGATGTTAACC | accuracy verification |
| Os19568704F | CCAATGTGAAGAAGGACCTG | accuracy verification |
| Os19568704R | CAGAGAGAGAGCTGAGAGAG | accuracy verification |
| Os22348468F | CTGGTGGGGTTTGGTTTTAA | accuracy verification |
| Os22348468R | AGGTTCTCACTCGAGGAAAA | accuracy verification |
| Os26447013F | GAGCCGCTGATGATTGGT | accuracy verification, variety identification |
| Os26447013R | AAAGCCGGTAGATTCCAAAC | accuracy verification, variety identification |
| Os30097070F | GATGGGATGAATCTTCTGCC | accuracy verification |
| Os30097070R | AGGAAAGCTTTGACAGTGTG | accuracy verification |
| Os30316456F | CTATGCCTAGGAAGTGCTCT | accuracy verification |
| Os30316456R | AGTTCAAAGTGCCTGAGTTC | accuracy verification |
| Os31234431F | CCTCCTTGCACTGTTTCTTG | accuracy verification |
| Os31234431R | CATTTCAGACTGTACGACCC | accuracy verification |
| Os31993051F | GATCGACTGATTCTACAGCG | accuracy verification |
| Os31993051R | AGGCTTCCTTTGATTCGAAC | accuracy verification |
| Os32275394F | CTCATGCTCATTGCTCTCAA | accuracy verification |
| Os32275394R | ACCCCAACCAAACAATTCTC | accuracy verification |
| Os753135F | GTTTTCGTCGTCGTCAAATT | accuracy verification |
| Os753135R | AAACGAAGGCTAGCAACAAT | accuracy verification |
| Os1241431F | GCATTGATAGGATCGCACTT | accuracy verification |
| Os1241431R | GTCGGGTTTTCCAAGTCTTT | accuracy verification |
| Os3811749F | CTCAAAAGCTGAGCCACC | accuracy verification |
| Os3811749R | GACACCTGACATGGAGTAGA | accuracy verification |
| Os5425050F | CATTCCTACATCAGCCATGG | accuracy verification |
| Os5425050R | TCAACGATTTTGGGCTTGAG | accuracy verification |
| Os6738491F | CATCTCCCTCAACTTCCTCT | accuracy verification |
| Os6738491R | ATCAGGTTGAACGTGAACTG | accuracy verification |
| Os16729065F | CAACCACCATATCAGCCAAA | accuracy verification |
| Os16729065R | CCTTGCTCTTAGTAATGGCC | accuracy verification |
| Os28396291F | AGCCATGCAAATGTTCTTCT | accuracy verification |
| Os28396291R | AGCACAATGTTGGTATGGAC | accuracy verification |
| Os31048249F | TTTCATACCTTGCACGTGAG | accuracy verification |
| Os31048249R | AGTACTGTCACTGCGAGTAA | accuracy verification |
| Os35693885F | CCTCTGATTCTGGCAAACAA | accuracy verification |
| Os35693885R | GCGAGGATGACTTGACATAC | accuracy verification |
| Os396921F | TCAAGGTCCTCTCTCTCTCT | accuracy verification |
| Os396921R | GAGAGAGAGAGAGCAAGTGT | accuracy verification |
| Os4396588F | CGGCATTGAAGTACTCGATG | accuracy verification |
| Os4396588R | CCTGCCTCTTACAACTTCAC | accuracy verification |
| Os19562176F | ATGGGAGAAAAAGCGAAGAC | accuracy verification |
| Os19562176R | GGCACAAAGGACAACAAAAG | accuracy verification |
| Os19827860F | CACTTACCTCTCAGGCTACA | accuracy verification, variety identification |
| Os19827860R | TACCTTTCCTCTTCGTCTCC | accuracy verification, variety identification |
| Os20231704F | GGAAGATGAGTCACTCCTCA | accuracy verification |
| Os20231704R | TAAGTTGTCAGCACATGTGG | accuracy verification |
| Os20421470F | GTGGAATAAGCTAAACGGCA | accuracy verification |
| Os20421470R | ACAGGCCATTACGAAAATGT | accuracy verification |
| Os23487855F | GCAACTAGCTAGAGCCAATC | accuracy verification |
| Os23487855R | TACAACCAGGATCCAAAGGA | accuracy verification |
| Os27564581F | GGTGCCTCTCTTCTCATACT | accuracy verification |
| Os27564581R | CATGCCCATGTTATTCCCAA | accuracy verification |
| Os30182639F | TTGGTCAGGTGGATTTCTT | accuracy verification |
| Os30182639R | TGTAAGGCAGTTAGGTCGTT | accuracy verification |
| Os31776143F | GTGTAAAGGGGAGCGATTTT | accuracy verification, variety identification |
| Os31776143R | GGGACCCAAAAACCTTGAAT | accuracy verification, variety identification |
| Os32834729F | AGAAGGAGAGAATAGTGGCC | accuracy verification |
| Os32834729R | ACTCTGGCTTGGTAATGACT | accuracy verification |
| Os437683F | GCCATTCAAGTTCTTGTCCA | accuracy verification |
| Os437683R | TGAGTAGGCTCGAAACAGAT | accuracy verification |
| Os3210838F | GCATTAGTTCGTTCGTCCCT | accuracy verification |
| Os3210838R | GAACACATAGATTCCCACCG | accuracy verification |
| Os3437745F | GCTCTTGAAAATCTCGGTGT | accuracy verification |
| Os3437745R | GTCTCCTTGCATGTACCTTC | accuracy verification |
| Os5360210F | AACCAACGCCGATGTTATAC | accuracy verification |
| Os5360210R | GAACAACATCGATGCGTCTA | accuracy verification |
| Os15598506F | TATGCGTTCTGTAATCCCCT | accuracy verification |
| Os15598506R | CTGCATAGAAAGGGTACGTG | accuracy verification |
| Os19869877F | AGCTGTCGTACAACATTCTG | accuracy verification |
| Os19869877R | CTGGAGCGTCACGAAGTA | accuracy verification |
| Os23741833F | GTCGAATTTGGAAGGGAGTC | accuracy verification |
| Os23741833R | CGAACGAAGACGACGATG | accuracy verification |
| Os23742196F | GAGTTTGTCATCTACCGGTG | accuracy verification |
| Os23742196R | GGGATGACATGTATGAGCAC | accuracy verification |
| Os26480678F | TCCTCATCCTTCTCAACCTC | accuracy verification |
| Os26480678R | CTCCATCTCCTTCCACCTC | accuracy verification |
| Os762930F | TGCATAGTATGGGGATCGTT | accuracy verification |
| Os762930R | GAGATATGCCTAGGAGGACC | accuracy verification |
| Os762888F | CCTTCATCTATGGCCGATTG | accuracy verification |
| Os762888R | GAGATATGCCTAGGAGGACC | accuracy verification |
| Os1524202F | TTCCTGCTGTTCTTGTTCAG | accuracy verification |
| Os1524202R | GAATTGCATTGGTGATCAGC | accuracy verification |
| Os2773379F | CCACTGTATGCAGATTTCCC | accuracy verification |
| Os2773379R | GCGCTTAATTTGTGATGTGG | accuracy verification |
| Os3432838F | TAGGAGACGAGGAACCTAGT | accuracy verification |
| Os3432838R | TACTTTGCTTTTCGCCTGAA | accuracy verification |
| Os4718416F | TGGGTTACCGTAGCACTTAT | accuracy verification, variety identification |
| Os4718416R | TAATTAGCGCGGCGAAATTA | accuracy verification, variety identification |
| Os6747948F | CGACTATGGTTTGTGTGTGT | accuracy verification |
| Os6747948R | CGGAGGAATAGGTGGTTTTG | accuracy verification |
| Os9332768F | CAGTCGTCCCATTTGTTCAT | accuracy verification |
| Os9332768R | TAAGCAGATTAGCGGCAAAG | accuracy verification |
| Os10385438F | AGTGGACACTGGTCTATCTG | accuracy verification |
| Os10385438R | AAAGAGAAGCCGAACACATC | accuracy verification |
| Os13037082F | TGCTTTGTCACCTTGTCAAA | accuracy verification |
| Os13037082R | CCTATGAGATGTTGTCGCAG | accuracy verification |
| Os12929694F | TGTTTCCAATTTCGCGTACT | accuracy verification |
| Os12929694R | ATGCATGCCAAACAAAAGTG | accuracy verification |
| Os17181894F | GTTAGTGCCGCTAAGTTGAA | accuracy verification |
| Os17181894R | CGACGGTAATATTTCCCGAC | accuracy verification |
| Os24340417F | GGAATACGTAGGCATGCAAT | accuracy verification |
| Os24340417R | TGCATTCGTGCTTAGAACAA | accuracy verification |
| Os25095178F | AATCACGCAGCAGATCAATT | accuracy verification |
| Os25095178R | AAACCACTTCACAACTCCAC | accuracy verification |
| Os27219152F | TGCCCATAGTAGTTTCTCGT | accuracy verification |
| Os27219152R | AATTAGTAGAGGGCTCGAGG | accuracy verification |
| Os27480778F | AACAACAACCAAGGCAAGAT | accuracy verification |
| Os27480778R | CTCGCCAACCCGATCAC | accuracy verification |
| Os28119449F | CACCGTTTGTTCATCTCCAT | accuracy verification, variety identification |
| Os28119449R | CTTAATCGCTCTGTGATGCA | accuracy verification, variety identification |
| Os30892084F | GATAACCACTGATCGGATGC | accuracy verification |
| Os30892084R | ACGAAATGCAAGTCTCTGTC | accuracy verification |
| Os1560741F | ATCAATTCGAAGTGGACGTG | accuracy verification |
| Os1560741R | ATCCACTGTAACCTACGGTT | accuracy verification |
| Os2832225F | GTGTCGCTTCAGATCATCTC | accuracy verification |
| Os2832225R | GGACACGAAAACGCATTACT | accuracy verification |
| Os4322489F | CTCAGCAAGTCCAAAATCCA | accuracy verification |
| Os4322489R | TGTGGATTCTGTCGAGAAGA | accuracy verification |
| Os9152479F | ATCTGAACCATTGTCCAAGC | accuracy verification |
| Os9152479R | CATATTGTGGGAGCACGTTC | accuracy verification |
| Os12918436F | CTTGCAATTGGATGAGACCA | accuracy verification |
| Os12918436R | AAGCAAGGGCACTCAATAAG | accuracy verification |
| Os25382707F | GGTGGAATGATCTTGCAGTT | accuracy verification |
| Os25382707R | ATAACTTGTGAAGGCACTCG | accuracy verification |
| Os28252688F | CTGAACCACGTGATGAGTTT | accuracy verification |
| Os28252688R | ATCTGAACTGAAGCTTGCAG | accuracy verification |
| Os29190825F | GATTGAACATGGCTCTGTGT | accuracy verification |
| Os29190825R | TAAGCTAATCTGCAAGGTGC | accuracy verification |
| Os3660969F | CACCAATGAATGAGGGTGTT | accuracy verification |
| Os3660969R | ATTCTCCATGCCACATGTTC | accuracy verification |
| Os4570420F | TACCATCGGTGAATGCTAGT | accuracy verification |
| Os4570420R | CTGAACCCTTCTAGCAGGTA | accuracy verification |
| Os5342240F | CTCAAGCTTGAAGGCATCAT | accuracy verification |
| Os5342240R | ATTAAACCATTGCCCCAGAC | accuracy verification |
| Os20367945F | CATCATCTCCACCATCACAC | accuracy verification |
| Os20367945R | CCTACGTGGCAGTCTAGTTA | accuracy verification |
| Os25216595F | GCAGATAAGAATGGTGCCAA | accuracy verification |
| Os25216595R | AGACAAAACGCTCCAAAACT | accuracy verification |
| Os25305375F | CATATCATCCAGCACAAGCA | accuracy verification, variety identification |
| Os25305375R | GCTAGATTGGCCGGTTAAAT | accuracy verification, variety identification |
| Os25889071F | GGGGCAAATGAACAGCTC | accuracy verification, variety identification |
| Os25889071R | ATAAATCTCCCGCCATTTCG | accuracy verification, variety identification |
| Os26508206F | CTCATCATCTTCTCCAACCG | accuracy verification |
| Os26508206R | CAGATGCAGCTAGCTTTTGA | accuracy verification |
| Os26726146F | GAGATTGCAACACGGTAAGT | accuracy verification, variety identification |
| Os26726146R | CTGATGAAGAAGACGACGAC | accuracy verification, variety identification |
| Os27991672F | TGCTCCCACAAGGAAAGT | accuracy verification |
| Os27991672R | GTAGGAGGAGAGCTCACATT | accuracy verification |
| Os7234952F | CGAGGAACACAATCTCGATC | accuracy verification |
| Os7234952R | ATGACCATACGGAGGCAC | accuracy verification |
| Os9619154F | CAAATTTGGCTAGTGGAGGG | accuracy verification |
| Os9619154R | CACGTGGGTCCGATTTTATT | accuracy verification |
| Os9802102F | CATGTCTTGGCTAGGATTCG | accuracy verification |
| Os9802102R | CGCTGACGTTGGGTATTTAT | accuracy verification |
| Os16422723F | TCTGTTCCCCACCTCTATTC | accuracy verification, variety identification |
| Os16422723R | TGGATGACTTCTTCCCAGTT | accuracy verification, variety identification |
| Os17348611F | CCAACTCATTGTCGACTTGT | accuracy verification |
| Os17348611R | CTTGTTGTCGCTAGATCCTG | accuracy verification |
| Os15672325F | TTGGATATGTTGACGTCTGC | accuracy verification |
| Os15672325R | TCAGATGGTCATCGACAAGA | accuracy verification |
| Os17076582F | TGCTTGTTGTTGGAATCGAT | accuracy verification |
| Os17076582R | GGGTGAACGAAATTACCGAA | accuracy verification |
| Os22137026F | CGTCTCTTGCTCTATTTCGG | accuracy verification |
| Os22137026R | ATTTACTGGATCTCCCTCCC | accuracy verification |
| Os576135F | ATGAAAGAAAGAGCCGCAAT | accuracy verification |
| Os576135R | ATATACCGTGCAATCAAGCA | accuracy verification |
| Os1229569F | GAGTACACCCTACATCCGAA | accuracy verification |
| Os1229569R | GGACAGTGTGGTAGAGAAGA | accuracy verification |
| Os2450044F | CGTCGATCACTTAGGGAAAC | accuracy verification |
| Os2450044R | CTAGCTCGCTGGATCTTCA | accuracy verification |
| Os6532244F | CACCATGACAAGGATGCTTC | accuracy verification |
| Os6532244R | TCATCGTTTGGAAGCGTATC | accuracy verification |
| Os7205693F | TGCCTGGTCTTCTCTGTTAA | accuracy verification |
| Os7205693R | TGTCTAATGAAAAGCGTGCA | accuracy verification |
| Os7963129F | GGTGACGAGTACTAGAGAGG | accuracy verification, variety identification |
| Os7963129R | AGATGCCACAAAGAAGGTTC | accuracy verification, variety identification |
| Os19179120F | TCCACATGCAAAGGTTTGAA | accuracy verification |
| Os19179120R | TCATGATTGCTACGACGAAC | accuracy verification |
| Os20801374F | AGACACGTTATGACACCAAC | accuracy verification |
| Os20801374R | TTAAGCAAAAGTCGATGGCA | accuracy verification |
| Os22222234F | TGCCATGGCTGATTTGATTT | accuracy verification |
| Os22222234R | CGGAGAACACATCACATCTC | accuracy verification |
| Os25241514F | TTTGAAGTTGCCATGGGTAG | accuracy verification |
| Os25241514R | TTCGTTTCTGATATGACCGC | accuracy verification |
| Os10606779F | GCACCAGGAATCTTGTCAAT | accuracy verification |
| Os10606779R | GTATCGAAGAAGACATGCCC | accuracy verification |
| Os16583663F | GTCGTCCTCGTTTCTGTAGTAGTTG | accuracy verification |
| Os16583663R | TCGATTACGGAGTGGGTGGTG | accuracy verification |
| Os17374158F | CCTTAGCCAGCATCTGAATC | accuracy verification |
| Os17374158R | TGCACTATTGTGGTTGACAG | accuracy verification |

**Table C. Primer sequences used for target breeding of *BADH2.***

| **Primer name** | **Sequence (5’ – 3’)** | **Purpose** |
| --- | --- | --- |
| Os20105487F | GTGTACGGGAGCGAAGAAGAT | targeted breeding of *BADH2* |
| Os20105487R | CTGCCACCTCTTGCTTGCTAT | targeted breeding of *BADH2* |
| Os20557750F | AGAACACCGAAATGACAACGA | targeted breeding of *BADH2* |
| Os20557750R | GGGTATAAGTTACGCAAATCACAA | targeted breeding of *BADH2* |
| Os5551891F | CTGTCATTTGCTGGGCTTTA | targeted breeding of *BADH2* |
| Os5551891R | ACATCCTGGCCTTTGCTCT | targeted breeding of *BADH2* |
| Os8723151F | CGGAACTCATACAAACGCTAC | targeted breeding of *BADH2* |
| Os8723151R | CGGAGTCGCTTCCAGTTAT | targeted breeding of *BADH2* |
| Os15110235F | TGGATGGAGTTGGACTGAAT | targeted breeding of *BADH2* |
| Os15110235R | GCAGCTCACTACTTCAACAA | targeted breeding of *BADH2* |
| Os17800476F | CTCAACTTCAAGGCGACCA | targeted breeding of *BADH2* |
| Os17800476R | ACGGGTCTTGATTCCTTGTAG | targeted breeding of *BADH2* |
| Os22293835F | GGAATGTCTTGGCAATGAA | targeted breeding of *BADH2* |
| Os22293835R | GCTTTAAGCGATCAAGGAGT | targeted breeding of *BADH2* |
| Os22845257F | AAGCGGTGAAGTGGATTGC | targeted breeding of *BADH2* |
| Os22845257R | CAGCCAGCACAGTGAGCAT | targeted breeding of *BADH2* |
| Os23659209F | TCCTACACTTTGCTCGCTTTC | targeted breeding of *BADH2* |
| Os23659209R | GCCTTGTTGTCGTAGTTCTCG | targeted breeding of *BADH2* |
| Os24896095F | GCAGAGCTAACAATCGAAAGG | targeted breeding of *BADH2* |
| Os24896095R | GACAAGAGGCGGACTGGAT | targeted breeding of *BADH2* |

**Table D. Summary of sequence data.**

|  | JP69 | Jiaoyuan5A |
| --- | --- | --- |
| Total reads | 16,970,386 | 23,550,928 |
| Unique mapped reads | 8,893,480 | 13,623,662 |
| Repeat mapped reads | 1,234,860 | 2,004,239 |
| Coverage (ratio)^a^ | 29,309,147 (47.7%) | 32,495,432 (51.8%) |
| Read length | 83bp | |
| High-quality markes(Density) | 82,878 SNPs (0.2 per kb)7,865InDels (0.02 per kb) | |
| Accuracy^b^ | 93% | |

**^a^** Number of positions covered by greater than or equal to eight reads.

^b^ Accuracy of high-quality markers.
